# Mediation of the Relationship between Maternal Phthalate Exposure and Preterm Birth by Oxidative Stress with Repeated Measurements across Pregnancy

**DOI:** 10.1289/EHP282

**Published:** 2016-06-28

**Authors:** Kelly K. Ferguson, Yin-Hsiu Chen, Tyler J. VanderWeele, Thomas F. McElrath, John D. Meeker, Bhramar Mukherjee

**Affiliations:** 1 Department of Environmental Health Sciences, and; 2 Department of Biostatistics, University of Michigan School of Public Health, Ann Arbor, Michigan, USA; 3 Department of Epidemiology and Biostatistics, Harvard T.H. Chan School of Public Health, Boston, Massachusetts, USA; 4 Division of Maternal-Fetal Medicine, Brigham and Women’s Hospital, Harvard Medical School, Boston, Massachusetts, USA

## Abstract

**Background:**

Mediation analysis is useful for understanding mechanisms and has been used minimally in the study of the environment and disease.

**Objective:**

We examined mediation of the association between phthalate exposure during pregnancy and preterm birth by oxidative stress.

**Methods:**

This nested case–control study of preterm birth (*n* = 130 cases, 352 controls) included women who delivered in Boston, Massachusestts, from 2006 through 2008. Phthalate metabolites and 8-isoprostane, an oxidative stress biomarker, were measured in urine from three visits in pregnancy. We applied four counterfactual mediation methods: method 1, utilizing exposure and mediator averages; method 2, using averages but allowing for an exposure–mediator interaction; method 3, incorporating longitudinal measurements of the exposure and mediator; and method 4, using longitudinal measurements and allowing for an exposure–mediator interaction.

**Results:**

We observed mediation of the associations between phthalate metabolites and all preterm birth by 8-isoprostane, with the greatest estimated proportion mediated observed for spontaneous preterm births specifically. Fully utilizing repeated measures of the exposure and mediator improved precision of indirect (i.e., mediated) effect estimates, and including an exposure–mediator interaction increased the estimated proportion mediated. For example, for mono(2-ethyl-carboxy-propyl) phthalate (MECPP), a metabolite of di(2-ethylhexyl) phthalate (DEHP), the percent of the total effect mediated by 8-isoprostane increased from 47% to 60% with inclusion of an exposure–mediator interaction term, in reference to a total adjusted odds ratio of 1.67 or 1.48, respectively.

**Conclusions:**

This demonstrates mediation of the phthalate–preterm birth relationship by oxidative stress, and the utility of complex regression models in capturing mediated associations when repeated measures of exposure and mediator are available and an exposure–mediator interaction may exist.

**Citation:**

Ferguson KK, Chen YH, VanderWeele TJ, McElrath TF, Meeker JD, Mukherjee B. 2017. Mediation of the relationship between maternal phthalate exposure and preterm birth by oxidative stress with repeated measurements across pregnancy. Environ Health Perspect 125:488–494; http://dx.doi.org/10.1289/EHP282

## Introduction

Understanding causal mechanism in observational studies of environmental exposure and complex disease is challenging. A useful approach may be to screen human populations for biomarkers of exposures as well as mechanistic intermediates and assess relationships with mediation analyses to aid in establishing biological pathways. We recently observed that urinary concentrations of phthalate metabolites were associated with increased odds of preterm birth (Ferguson et al. 2014). Phthalate metabolites are indicative of exposure to phthalate diesters which are found ubiquitously in the environment in plastics, personal care products, and medications (ATSDR 2001, 2002). Exposure to these compounds during pregnancy has been linked to preterm birth in other previous studies as well, although some results are conflicting (Adibi et al. 2009; Meeker et al. 2009; Whyatt et al. 2009; Wolff et al. 2008). We hypothesized that this relationship may be mediated by phthalate-induced maternal oxidative stress. Our previous work circumstantially supports this hypothesis; we have established that urinary phthalate metabolites are associated with an increase in urinary 8-isoprostane, a biomarker of oxidative stress (Ferguson et al. 2015b), and that furthermore urinary 8-isoprostane is associated with an increased risk of preterm birth (Ferguson et al. 2015a). In the present analysis, we sought to quantify and test the role of 8-isoprostane in the relationship between maternal phthalate exposure during pregnancy and prematurity using a mediation analysis within a causal framework.

Mediation analysis has been used in social and epidemiological research for decades to understand causal pathways, biological mechanisms, and to design policy interventions (Pearl 2014). Its development originated in structural equation modeling, and with the work of Baron and Kenny (1986) became widely used in the context of linear models. Application of a counterfactual framework (Rubin 1974) has further provided a strong theoretical basis for causal inference from mediation analysis by precisely defining the necessary assumptions. This framework has also facilitated natural extension of mediation analysis to more complex models that include exposure–mediator interaction, nonlinear terms, and longitudinal data (Valeri and VanderWeele 2013; VanderWeele and Tchetgen Tchetgen 2014; VanderWeele and Vansteelandt 2010).

The use of mediation analysis in environmental and exposure epidemiology has been infrequent and more recent. For example, in the Normative Aging Study partial mediation of the relationship between black carbon particulate exposure and increased fibrinogen levels was observed through epigenetic modifications (Bind et al. 2014). The limited application of mediation analysis is surprising, because many studies in environmental epidemiology measure markers of intermediate biological changes (e.g., hormone levels, epigenetic modifications, and inflammatory cytokines) in addition to examining exposure and disease outcomes.

**Figure 1:**
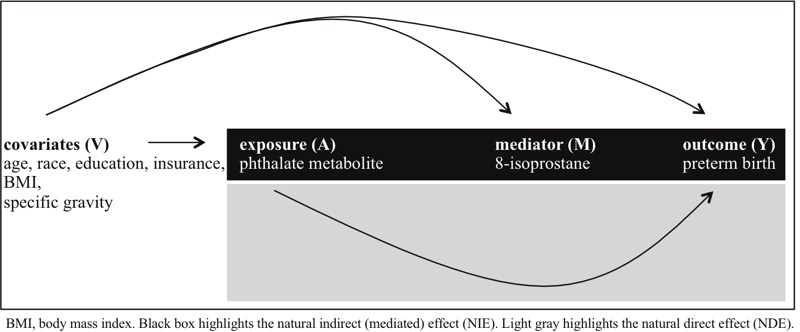
Simple conceptual model for mediation analysis in the context of the present study (adapted from VanderWeele and Tchetgen Tchetgen 2014).

The goal of the present study was to examine causal mediation in an observational study of preterm birth. Specifically, we investigated mediation of the relationship between exposure to phthalates, as indicated by urinary phthalate metabolites, and preterm birth by oxidative stress, as indicated by urinary 8-isoprostane. Within the causal framework, we investigated the natural direct effect (NDE) and the natural indirect (i.e., mediated) effect (NIE), as illustrated in Figure 1. Statistically speaking, the NDE refers to the change in the odds of preterm birth (Y) in association with a defined change in the urinary phthalate concentration (A; e.g., from a to a*) while holding the urinary 8-isoprostane concentration (M) at the level it would have naturally been at with A set at the original level (e.g., a). The NIE refers to the change in the odds of preterm birth (Y) when urinary phthalate concentration is held at a specific level (e.g., a) and 8-isoprostane is set at what it would have naturally been at for the defined change in urinary phthalate concentration (e.g., a*) (Pearl 2014).

In addition to applying this counterfactual framework to allow for causal interpretation of our results, we wanted to appropriately use the repeated measures available in this data set to fully capture mediated effects. There are very few empirical examples of mediation analysis with longitudinal data in the literature (MacKinnon 2012), and none in a causal framework with time-varying exposure and mediators and a non–time-varying binary end point. Thus, we applied four analytic strategies to this study using recent advancements in the causal inference literature for mediation analysis, using the counterfactual framework. These include method 1, an approach utilizing exposure and mediator averages; method 2, using averages but allowing for an exposure–mediator interaction; method 3, a longitudinal approach utilizing repeated measures of exposure and mediator; and method 4, using repeated measures and allowing for an exposure–mediator interaction.

## Methods

### Study Population

The study population has been described in detail previously (Ferguson et al. 2014). Briefly, mothers included in this nested case–control study were selected from a prospective birth cohort examining predictors of preeclampsia in women who delivered at the Brigham and Women’s Hospital in Boston, Massachusetts, from 2006 through 2008. The present study, designed with the primary purpose of examining phthalate exposure in relation to preterm birth, included 130 women who delivered preterm as well as 352 randomly selected controls. Participants provided written informed consent, and institutional review board approval was obtained from Brigham and Women’s Hospital as well as the University of Michigan. Preterm birth was defined as delivery before 37 weeks gestation based on last menstrual period with verification by first-trimester ultrasound (hereafter denoted all preterm birth). We also examined spontaneous preterm births alone (delivery < 37 weeks gestation preceded by spontaneous preterm labor and/or preterm premature rupture of membranes), because we had previously observed strongest associations within this subgroup and we hypothesized that oxidative stress would play a more important role in this pathway based on biologic plausibility (Ferguson et al. 2014).

Urine samples were available for analysis of phthalate and oxidative stress biomarkers from up to four visits per subject (mean, 3.52 visits per subject) across gestation, at median 10, 18, 26, and 35 weeks gestation. For the present analysis, we used measures from visits 1–3 only because many of the preterm cases had delivered by the 35-week visit and those measurements may be biased. Demographic characteristics that were included in regression models in previous analyses within this population included maternal age at visit 1, race/ethnicity (white, African American, other), education level (high school, technical school, junior college/some college, college graduate), health insurance provider (private vs. public), and prepregnancy body mass index (BMI; < 25 kg/m^2^, 25 to < 30 kg/m^2^, ≥ 30 kg/m^2^). These covariates were included in each model for the present analysis; categorical variables were modeled with the following reference levels: race/ethnicity (white), education level (high school), health insurance provider (private), and prepregnancy BMI (< 25 kg/m^2^). In addition, we performed a sensitivity analysis treating the most frequently occurring categorical variables (white, college graduate, private health insurance, prepregnancy BMI < 25 kg/m^2^) as reference levels for comparison.

### Urinary Exposure and Mediator Biomarkers

NSF International (Ann Arbor, MI) measured nine urinary phthalate metabolites by high performance liquid chromatography and tandem mass spectrometry as described previously (Ferguson et al. 2014; Lewis et al. 2013). All metabolites were detected in > 95% of urine samples, and levels below the limit of detection were kept as is if reported and otherwise replaced by the limit of detection divided by the square root of 2. In addition to individual metabolites, a summed measure of di(2-ethylhexyl) phthalate (DEHP) metabolites, including mono(2-ethylhexyl) phthalate (MEHP), mono(2-ethyl-5-hydroxyhexyl) phthalate (MEHHP), mono(2-ethyl-5-oxohexyl) phthalate (MEOHP), and mono(2-ethyl-5-carboxypentyl) phthalate (MECPP), was created based on nanomolar concentrations. Because DEHP metabolites are highly correlated, and the strongest associations observed with all preterm birth were for MEHP and MECPP, MEHHP and MEOHP were not examined separately in this analysis. Cayman Chemical (Ann Arbor, MI) measured total 8-isoprostane in affinity purified urine samples via enzyme immunoassay, and detection was to 3.9 pg/mL. Levels below the limit of detection (4%) for 8-isoprostane concentrations were treated the same as phthalate metabolites. In addition, to adjust for urinary dilution, specific gravity was measured in all samples at the time of phthalate analysis with a digital handheld refractometer (Atago Co., Ltd., Tokyo, Japan). We did not correct phthalate metabolite or 8-isoprostane concentrations to specific gravity measures, but instead models were adjusted for specific gravity to achieve more precise estimates of urine biomarkers. Urinary phthalate metabolites and 8-isoprostane measurements are logarithm-transformed and then standardized with mean of 0 and standard deviation of 1 throughout the mediation analysis for better interpretability and comparability across different phthalates.

Of the overall 482 subjects, 357 had measures of phthalate metabolites and 8-isoprostane available from all three study visits. One hundred twenty-five subjects had at least one missing 8-isoprostane measurement (*n* = 8, 61, and 73 for visits 1–3, respectively) and 118 had at least one missing urinary phthalate metabolite measurement (*n* = 3, 60, 70 for visits 1–3, respectively). For time points where 8-isoprostane or phthalate metabolites were missing, we applied single imputation of the geometric average of that subject’s urinary concentrations from other visits. We additionally performed sensitivity analyses to compare results across three methods of treating missing data: *a*) subjects with all exposure mediator measures available or a complete case analysis; *b*) data with imputation based on subject-specific average; and *c*) data with imputation based on average of all subjects at that visit.

### Mediation Methods

Traditional mediation analysis enables the researcher to identify the proportion of a statistical relationship between exposure A and outcome Y that occurs through a change in the mediator M using a sum or product coefficient method. The counterfactual approach to mediation analysis differs from the traditional approaches developed by Baron and Kenny (1986) in that it clarifies the assumptions that allow for causal interpretation of results and enables extension to more sophisticated and general data structures and models (Pearl 2014). The underlying assumptions are key to application of the counterfactual framework. Most important, this includes the assumption of no unmeasured confounding within *a*) the exposure–outcome relationship; *b*) the mediator–outcome relationship; and *c*) the exposure–mediator relationship. Furthermore, the analysis assumes that none of the mediator–outcome confounders are affected by exposure. Our ability to meet these assumptions in the present example is addressed in the Discussion section.

The statistical notation for the models is as follows: Let *A*(*t*) represent the logarithm of urinary phthalate metabolites at visit *t*, *A* = [*A*(1), *A*(2), *A*(3)], and *A* ^^—^^ represent the average of the log-transformed phthalate metabolite concentrations across the three visits; *M*(*t*) represent the logarithm of the mediator 8-isoprostane at visit *t*, *M* = [*M*(1), *M*(2), *M*(3)], and *M* ^^—^^ represent the average of the log-transformed concentrations of 8-isoprostane across the three visits; *Y* represent the outcome, all or spontaneous preterm birth; *V* represent the set of time-invariant baseline covariates listed above; and *L*(*t*) represent the only time-varying covariate, urinary specific gravity, at visit *t*, *L* = [*L*(1), *L*(2), *L*(3)], and *L* ^^—^^ represent the average across the three visits. *A* ^^—^^ and *M* ^^—^^ have been standardized to have a mean of zero and a standard deviation of one for ease of interpretability. Finally, upper-case letters represent the random variables and the corresponding lower-case letters represent possible realizations of the random variables. All statistical analyses were performed using R (version 3.1.0; R Project for Statistical Computing). A more detailed and complete description of the subsequent statistical models, methods, and corresponding R code are available in Section S1. Steps for Mediation Analysis with Longitudinal Exposure and Mediator with a Binary Outcome.

***Method 1: utilizing exposure and mediator averages.*** We first applied a simple counterfactual approach toward performing a mediation analysis on these data by creating subject-specific averages of the time-varying exposure, mediator, and time-varying covariate variables. The two statistical models used are as follows:

*Model 1*: *logit*[*P*(*Y* = 1|*a*, *m*, ***v***, *l*)] = β*_y0_* + β*_ya_a̅* + β*_ym_m̅* + **β*^T^_yv_v*** + β*_yl_l̅*

*Model 2: E*[*M̅*|*a*, ***v***, *l*] = β*_m0_* + β*_ma_a̅* + **β*^T^_mv_v*** + β*_ml_l̅*

In model 2 we applied inverse probability weightings to account for the nested case–control study design. The estimates of NDE and NIE are based on the expressions in the appendix of VanderWeele and Vansteelandt (2010), and the standard errors are obtained by bootstrap. The total effect can be expressed as the product of NDE and NIE in the original beta coefficient scale for linear models. On the difference scale for log odds ratios, the total effect can be defined as the sum of NDE and NIE and the proportion mediated is then 
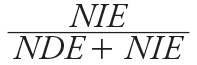
.

***Method 2: utilizing exposure and mediator averages with interaction.*** Our second approach was to employ the same counterfactual framework, but allow for an interaction between the exposure and mediator averages. The NDE and NIE can be estimated in the same fashion as the previous method from the following two models:

*Model 3*: *logit*[*P*(*Y* = 1|*a, m*, ***v***, *l*)] = β*_y_*_0_ + β*_ya_a̅* + β*_ym_m̅* + β*_yi_a̅m̅* + **β*^T^_yv_v*** + β*_yl_l̅*

*Model 4:* *E*[*M̅*|*a*, ***v***, *l*] = β*_m_*_0_ + β*_ma_a̅* + **β*^T^_mv_v*** + β*_ml_l̅*

The interaction between urinary phthalate metabolite and urinary 8-isoprostane is taken into account in model 3. As in the previous method, for model 4 we applied inverse probability weightings to adjust for the case–control study design. Because the estimate of NDE depends on the level of the covariates, we fixed the continuous covariates at their arithmetic means and set the categorical covariates at their reference levels for this and subsequent analysis.

***Method 3: longitudinal approach utilizing repeated measures of exposure and mediator.*** Our third approach was to apply the randomized interventional analogue of natural direct and indirect effects defined by VanderWeele and Tchetgen Tchetgen (2014) in the longitudinal setting. Using longitudinal detail entails additional assumptions. These include *a*) that no time-varying confounder is affected by prior exposure or mediator; and *b*) that at each time point the exposure–mediator, exposure–outcome, and mediator–outcome relationships conditional on the past are unconfounded. Under the latter assumption the randomized interventional analogues of natural direct and indirect effects can be derived from the g-formula (Robins 1986). When there are no time-varying confounders, this g-formula reduces to the longitudinal version of Pearl’s mediation formula (2014). There are no time-varying confounders in our scenario, because specific gravity is not a confounder of the exposure–outcome relationship. Thus, we applied the latter formula to estimate the natural direct and indirect effects using marginal structural models (Robins et al. 2000). The marginal structural models are as follows:

*Model 5*: *logit*[*P*(*Y_am_* = 1 / *a, m,* ***v***, *l*)] = β*_y_*_0_ + β*_ya_cum*(*a*) + β*_ym_cum*(*m*) + **β*^T^_yv_v*** + β***_yl_*** *l̅*

*Model 6*: *E*[*M_a_*(*t*)|*a*(1), …, *a*(*t*), ***v***, *l*(*t*)] = β*_m_*_0_(*t*) + β*_ma_*(*t*)*avg*[*a*(*t*)] + **β*^T^_mv_*(** *t* **)*v*** + β***_ml_*(*t*)*l*(** *t* **)** for *t* = 1, 2, 3

where *cum* is the cumulative total of the measures across all time points, and *avg*[*a*(*t*)] is the average of *a* (*exposure*), up to and including time point *t*. The conditional model can be interpreted as a structural model if there is no time-dependent confounding. Inverse probability weighting to adjust for the case-control study design are applied to each of the three regressions in model 6. The direct and indirect effects are statistically defined in a similar fashion to the second approach, except that change from one exposure history to another exposure history in time-varying sense rather than change from one level of exposure to another level now needs to be specified. The expressions for direct and indirect effects are detailed in Section S1. Steps for Mediation Analysis with Longitudinal Exposure and Mediator with a Binary Outcome; Method 3 - Counterfactual Approach with Longitudinal Exposure and Longitudinal Mediator without Interaction (VanderWeele and Tchetgen Tchetgen 2014). The scenario constructed for interpreting the mediation effect here corresponds to an ln-unit increase in exposure across all three time points. The NDE, NIE, and proportion mediated are then expressed accordingly and the corresponding standard errors are obtained by bootstrap for inference purposes.

***Method 4: longitudinal approach utilizing repeated measures of exposure and mediator with interaction.*** Our fourth approach was to extend the third approach to account for an exposure–mediator interaction, in the same way that Method 2 extends Method 1. More detailed descriptions are provided in Section S1. Steps for Mediation Analysis with Longitudinal Exposure and Mediator with a Binary Outcome; Method 4 - Counterfactual Approach with Longitudinal Exposure and Longitudinal Mediator with Interaction (VanderWeele and Tchetgen Tchetgen 2014). Similarly, we applied a g-formula approach to estimate the direct and indirect effects using marginal structural models and inverse probability of treatment weighting (Robins et al. 2000) under the same set of assumptions. The marginal structural models are:

*Model 7*: *logit*[*P*(*Y_am_* = 1*|a*, *m*, ***v***, *l*)] = β*_y_*_0_ + β*_ya_cum*(*a*) + β*_ym_cum*(*m*) + β*_yi_cum*(*x*)*cum*(*m*) + **β*^T^_yv_v*** + β***_yl_*** *l̅*

*Model 8*:*E*[*M_a_*(*t*)|*a*(1), …, *a*(*t*), ***v***, *l*(*t*)] = β*_m_*_0_(*t*) + β*_ma_(t*)*avg*[*a*(*t*)] + **β*^T^_mv_*(** *t* **)*v*** + β***_ml_*(*t*)*l*(** *t* **)** for *t* = 1, 2, 3

Error Terms in Model 8 ***ϵ*** = [*ϵ*(1), *ϵ*(2), *ϵ*(3)]*^T^* ∼ *MVN*(**0**, σ^2^∑),

where Σ can be an arbitrary 3 × 3 positive-definite matrix with 1s along the diagonal. Model 8 is the same as Model 6 except for an additional assumption, that the three errors terms from the three regressions follow a zero-mean multivariate normal distribution with common variance and unstructured correlation. The conditional model can be interpreted as a structural model if there is no time-dependent confounding. The time-varying coefficients are jointly estimated using generalized least squares. The reason for assuming a joint correlation structure across the three regression residuals is to obtain the estimated variance of Σ^3^ *_t_*_= 1_ *M*(*t*), a necessary quantity for estimating NDE in the scenario with exposure–mediator interaction. The inverse probability weighting to adjust for the case–control study design is applied to model 8 as before. The expressions for direct and indirect effects and their derivations are provided in Section S1. Steps for Mediation Analysis with Longitudinal Exposure and Mediator with a Binary Outcome; Method 4 - Counterfactual Approach with Longitudinal Exposure and Longitudinal Mediator with Interaction (VanderWeele and Tchetgen Tchetgen 2014). We used the same scenario as constructed in Method 3 where we assessed the direct effect, indirect effect, and proportion mediated based on unit change in exposure level at each time point and subsequently drew inference based on the standard errors obtained by bootstrap.

## Results

Beta coefficients and standard errors from regression models for each approach are similar to results observed previously (Tables S1–S4; Ferguson et al. 2014, 2015a, 2015b). Urinary phthalate metabolites were positively associated with preterm birth, and associations were stronger for spontaneous preterm birth specifically. Effect estimates were greatest in magnitude, and confidence intervals most precise, for associations with DEHP metabolites or MBP. Also, 8-isoprostane was positively associated with preterm and particularly spontaneous preterm birth, and with urinary phthalate metabolites.

Mediation results for Methods 1–4 are shown in Tables 1–4, respectively. Each table shows results for all and spontaneous preterm births separately. Effect estimates represent ln-odds ratios in association with a 1 standard deviation change (from mean – 1 to the mean) in exposure average (Methods 1–2) or across the three study visits (Methods 3–4). For all preterm birth, mediation analysis was performed for MEHP, MECPP, ΣDEHP, and mono-*n-*butyl phthalate (MBP) only, because associations in regression models were not statistically significant for other metabolites.

**Table 1:** Effect estimates*^a^* (95% confidence intervals) with ln-unit increase in exposure and estimated percent mediated, calculated from regression estimates and standard errors generated from models 1–2 (Table S1) under Method 1: Counterfactual approach utilizing exposure and mediator averages.

All preterm	Natural direct effect (95% CI)	Natural indirect effect (95% CI)	Total effect (95% CI)	Estimated percent mediated^*b*^
MEHP	0.264 (0.009, 0.541)	0.062 (0.011, 0.147)	0.325 (0.075, 0.611)	19
MECPP	0.264 (0.009, 0.526)	0.114 (0.045, 0.226)	0.378 (0.134, 0.647)	30
∑DEHP	0.207 (–0.061, 0.484)	0.099 (0.036, 0.204)	0.307 (0.042, 0.595)	32
MBP	0.170 (–0.198, 0.490)	0.107 (0.039, 0.232)	0.277 (–0.070, 0.614)	39

Spontaneous preterm	Natural direct effect (95% CI)	Natural indirect effect (95% CI)	Total effect (95% CI)	Estimated percent mediated^*b*^
MEHP	0.476 (0.108, 0.922)	0.133 (0.012, 0.303)	0.609 (0.236, 1.092)	22
MECPP	0.274 (–0.094, 0.684)	0.287 (0.152, 0.510)	0.561 (0.201, 1.018)	51
∑DEHP	0.339 (–0.032, 0.772)	0.242 (0.109, 0.459)	0.581 (0.210, 1.066)	42
MBzP	0.288 (–0.143, 0.756)	0.140 (–0.005, 0.335)	0.428 (0.001, 0.919)	33
MBP	0.303 (–0.228, 0.675)	0.248 (0.097, 0.526)	0.551 (0.061, 0.999)	45
MiBP	0.274 (–0.149, 0.775)	0.179 (0.006, 0.439)	0.453 (0.032, 1.010)	39
MEP	0.204 (–0.155, 0.589)	0.160 (0.010, 0.373)	0.364 (–0.009, 0.781)	44
MCPP	0.247 (–0.175, 0.636)	0.215 (0.068, 0.433)	0.462 (0.044, 0.910)	47
Abbreviations: CI, confidence interval; MBzP, mono-benzyl phthalate; MCPP, mono(3-carboxypropyl) phthalate; MEP, mono-ethyl phthalate; MiPB; mono-isobutyl phthalate. ^***a***^The natural direct effect, natural indirect effect, and total effect reflect the natural log odds ratios. ^***b***^Percent mediated = natural indirect effect/(natural direct effect + natural indirect effect) × 100.				

We observed significant mediation (i.e., NIE with confidence intervals that did not include zero) for the relationship between urinary phthalate metabolites and all preterm birth as well as spontaneous preterm birth by 8-isoprostane. Significant NIE were observed across all Methods, and we consistently estimated a greater proportion mediated for relationships with spontaneous versus all preterm births. In regard to specific phthalate metabolites, the greatest estimated proportion of the preterm birth relationship mediated by 8-isoprostane was for MBP across all methods. For spontaneous preterm birth, the greatest estimated proportion mediated for each approach was either for MECPP or MBP.

Through applying the four different mediation approaches, we were able to compare *a*) the effect of incorporating an exposure–mediator interaction and *b*) the effect of using longitudinal exposure and mediator trajectories instead of reducing repeated measurements to an average. For comparison of results across mediation methods in the text, we present results for MECPP and MBP only because we observed the greatest mediation by 8-isoprostane for models of these metabolites in relation to spontaneous preterm birth (see other results in Tables 1–4). Incorporating interaction into mediation models generally increased the estimated percent mediated. In methods with exposure and mediator averages (Methods 1 and 2), estimated percent mediated increased from 51% to 61% for MECPP and from 45% to 70% for MBP. In methods with exposure and mediator treated longitudinally (Methods 3 and 4), estimated percent mediated increased from 47% to 60% for MECPP and from 39% to 81% for MBP.

Using repeated measures of exposure and mediator variables did not have a clear impact on the estimated proportion mediated. However, it did affect the precision of NIE (i.e., mediated effect) as evidenced by narrowing of the confidence intervals for these estimates. When methods without interaction terms (Methods 1 and 3) were compared, the width of the NIE confidence intervals for spontaneous preterm birth decreased from 0.358 to 0.314 for MECPP and from 0.429 to 0.333 for MBP. When methods with interaction terms (Methods 2 and 4) were compared, the width of the NIE confidence intervals for spontaneous preterm birth decreased from 0.369 to 0.307 for MECPP and from 0.436 to 0.342 for MBP.

In sensitivity analyses, we examined the impact of using imputation by subject-specific average for exposure and mediator values using the most general Method 4. (Method 4 is considered the most general modeling framework because it includes multiple measures of exposure and mediator and no exposure by mediator interaction. Methods 1–3 can all be viewed as special cases of Method 4, because they are all reduced versions of that method.) As alternatives to imputation with subject-specific exposure or mediator averages, we estimated NIE and NDE when imputing by *a*) using only subjects with available measures (complete case analysis); or *b*) using the population averages of those measures. This comparison showed that the estimated percent mediated was attenuated in models with imputation by population averages and that our approach with single imputation gave point estimates that were most consistent with those observed in models using subjects without missing values (Table S5).

Additionally, in Methods 2 and 4 with interaction we examined the impact of changing reference categories for categorical covariates to those observed most frequently in the study population. The estimated proportion mediated was similar across these choices (data not shown). Table 2. Table 3. Table 4.

**Table 2:** Effect estimates (95% confidence intervals) with ln-unit increase in exposure and estimated percent mediated calculated from regression estimates and standard errors generated from models 3–4 (Table S2) under Method 2: Counterfactual approach utilizing exposure and mediator averages with interaction.

All preterm	Natural direct effect^*a,b*^ (95% CI)	Natural indirect effect^*a*^ (95% CI)	Total effect^*a*^ (95% CI)	Estimated percent mediated^*c*^
MEHP	0.276 (0.012, 0.575)	0.070 (0.014, 0.158)	0.346 (0.081, 0.651)	20
MECPP	0.264 (–0.013, 0.523)	0.114 (0.047, 0.233)	0.378 (0.124, 0.650)	30
∑DEHP	0.215 (–0.075, 0.506)	0.104 (0.041, 0.221)	0.319 (0.043, 0.625)	33
MBP	0.169 (–0.233, 0.516)	0.106 (0.041, 0.237)	0.276 (–0.096, 0.643)	39

Spontaneous preterm	Natural direct effect^*a,b*^ (95% CI)	Natural indirect effect^*a*^ (95% CI)	Total effect^*a*^ (95% CI)	Estimated percent mediated^*c*^
MEHP	0.488 (–0.010, 1.034)	0.133 (0.013, 0.309)	0.621 (0.131, 1.214)	21
MECPP	0.187 (–0.423, 0.639)	0.288 (0.156, 0.525)	0.475 (–0.068, 0.972)	61
∑DEHP	0.282 (–0.282, 0.784)	0.242 (0.110, 0.465)	0.524 (–0.008, 1.076)	46
MBzP	0.223 (–0.311, 0.718)	0.142 (–0.007, 0.341)	0.365 (–0.159, 0.881)	39
MBP	0.107 (–0.747, 0.552)	0.251 (0.104, 0.540)	0.358 (–0.412, 0.828)	70
MiBP	0.211 (–0.382, 0.793)	0.180 (0.002, 0.446)	0.391 (–0.180, 1.032)	46
MEP	0.172 (–0.361, 0.606)	0.160 (0.013, 0.364)	0.332 (–0.191, 0.804)	48
MCPP	0.170 (–0.388, 0.587)	0.219 (0.073, 0.443)	0.389 (–0.138, 0.850)	56
Abbreviations: CI, confidence interval; MBzP, mono-benzyl phthalate; MCPP, mono(3-carboxypropyl) phthalate; MEP, mono-ethyl phthalate; MiPB; mono-isobutyl phthalate. ^***a***^The natural direct effect, natural indirect effect, and total effect reflect the natural log odds ratios and are measured based on logarithm-transformed exposure changed from the mean level minus 1 standard deviation to the mean level. ^***b***^The natural direct effect is conditional on the level of the covariates. Continuous covariates are fixed at their arithmetic means and categorical covariates are set at reference levels (white race/ethnicity, high school level education, private/health maintenance organization/self-pay health insurance, and BMI < 25 kg/m^2^). ^***c***^Percent mediated = natural indirect effect/(natural direct effect + natural indirect effect) × 100.				

**Table 3:** Effect estimates (95% confidence intervals) with ln-unit increase in exposure and estimated percent mediated calculated from regression estimates and standard errors generated from models 5–6 (Table S3) under Method 3: Longitudinal approach utilizing repeated measures of exposure and mediator.

All preterm	Natural direct effect^*a*^ (95% CI)	Natural indirect effect^*a*^ (95% CI)	Total effect^*a*^ (95% CI)	Estimated percent mediated^*b*^
MEHP	0.264 (0.011, 0.542)	0.054 (0.012, 0.125)	0.317 (0.069, 0.600)	17
MECPP	0.264 (0.024, 0.528)	0.100 (0.040, 0.195)	0.364 (0.131, 0.634)	27
∑DEHP	0.207 (–0.059, 0.480)	0.085 (0.031, 0.172)	0.292 (0.035, 0.568)	29
MBP	0.170 (–0.204, 0.494)	0.089 (0.032, 0.187)	0.259 (–0.102, 0.587)	34

Spontaneous preterm	Natural direct effect^*a*^ (95% CI)	Natural indirect effect^*a*^ (95% CI)	Total effect^*a*^ (95% CI)	Estimated percent mediated^*b*^
MEHP	0.486 (0.107, 0.951)	0.118 (0.013, 0.262)	0.604 (0.220, 1.089)	20
MECPP	0.271 (–0.091, 0.675)	0.243 (0.125, 0.439)	0.514 (0.166, 0.955)	47
∑DEHP	0.339 (–0.026, 0.784)	0.200 (0.087, 0.390)	0.540 (0.176, 1.019)	37
MBzP	0.284 (–0.142, 0.747)	0.147 (0.022, 0.332)	0.431 (0.010, 0.921)	34
MBP	0.298 (–0.245, 0.672)	0.194 (0.068, 0.401)	0.492 (–0.012, 0.910)	39
MiBP	0.273 (–0.134, 0.753)	0.168 (0.011, 0.394)	0.441 (0.026, 0.973)	38
MEP	0.200 (–0.160, 0.571)	0.172 (0.044, 0.360)	0.372 (0.006, 0.782)	46
MCPP	0.251 (–0.172, 0.644)	0.189 (0.064, 0.369)	0.440 (0.016, 0.883)	43
Abbreviations: CI, confidence interval; MBzP, mono-benzyl phthalate; MCPP, mono(3-carboxypropyl) phthalate; MEP, mono-ethyl phthalate; MiPB; mono-isobutyl phthalate. ^***a***^The natural direct effect, natural indirect effect, and total effect reflect the natural log odds ratios. ^***b***^Percent mediated = natural indirect effect/(natural direct effect + natural indirect effect) × 100.				

**Table 4:** Effect estimates (95% confidence intervals) with ln-unit increase in exposure and estimated percent mediated calculated from regression estimates and standard errors generated from models 7–8 (Table S4) under Method 4: Longitudinal approach utilizing repeated measures of exposure and mediator with interaction.

All preterm	Natural direct effect^*a,b*^ (95% CI)	Natural indirect effect^*a*^ (95% CI)	Total effect^*a*^ (95% CI)	Estimated percent mediated^*c*^
MEHP	0.299 (0.033, 0.594)	0.077 (0.033, 0.142)	0.376 (0.103, 0.692)	20
MECPP	0.264 (–0.006, 0.528)	0.094 (0.041, 0.183)	0.358 (0.096, 0.637)	26
∑DEHP	0.225 (–0.063, 0.505)	0.086 (0.037, 0.166)	0.310 (0.027, 0.607)	28
MBP	0.168 (–0.230, 0.549)	0.112 (0.054, 0.229)	0.280 (–0.081, 0.684)	40

Spontaneous preterm	Natural direct effect^*a,b*^ (95% CI)	Natural indirect effect^*a*^ (95% CI)	Total effect^*a*^ (95% CI)	Estimated percent mediated^*c*^
MEHP	0.501 (–0.052, 1.097)	0.147 (0.058, 0.293)	0.648 (0.095, 1.288)	23
MECPP	0.158 (–0.525, 0.610)	0.235 (0.126, 0.433)	0.392 (–0.235, 0.885)	60
∑DEHP	0.268 (–0.372, 0.804)	0.202 (0.100, 0.389)	0.469 (–0.132, 1.048)	43
MBzP	0.222 (–0.348, 0.695)	0.282 (0.147, 0.520)	0.504 (–0.048, 1.062)	56
MBP	0.049 (–0.933, 0.495)	0.212 (0.101, 0.443)	0.261 (–0.635, 0.740)	81
MiBP	0.210 (–0.422, 0.787)	0.327 (0.159, 0.601)	0.537 (–0.072, 1.214)	61
MEP	0.164 (–0.419, 0.603)	0.238 (0.114, 0.440)	0.401 (–0.185, 0.927)	59
MCPP	0.141 (–0.498, 0.588)	0.199 (0.091, 0.377)	0.340 (–0.264, 0.826)	59
Abbreviations: CI, confidence interval; MBzP, mono-benzyl phthalate; MCPP, mono(3-carboxypropyl) phthalate; MEP, mono-ethyl phthalate; MiPB; mono-isobutyl phthalate. ^***a***^The natural direct effect, natural indirect effect, and total effect reflect the natural log odds ratio and are measured based on logarithm-transformed exposure changed from the mean level minus 1 standard deviation to the mean level across three visits. ^***b***^The natural direct effect is conditional on the level of the covariates. Continuous covariates are fixed at their arithmetic means and categorical covariates are set at reference levels (white race/ethnicity, high school level education, private/health maintenance organization/self-pay health insurance, and BMI < 25 kg/m^2^). ^***c***^Percent mediated = natural indirect effect/(natural direct effect + natural indirect effect) × 100.				

## Discussion

We previously observed that urinary phthalate metabolites measured at multiple time points during pregnancy were associated with increases in urinary 8-isoprostane, a systemic biomarker of oxidative stress, and that both were associated with increased risk of all and especially spontaneous preterm birth. In the present mediation analysis, we statistically demonstrated within a causal framework that the relationship between phthalate exposure and spontaneous preterm birth is mediated in part by phthalate-induced oxidative stress, which has not been elucidated clearly in human or animal studies previously. The higher estimated proportion mediated by 8-isoprostane observed in models of spontaneous preterm births alone indicates that the oxidative stress pathway may be particularly relevant for this subtype.

The latter finding has biologic plausibility based on what is known about the role of oxidative stress and prematurity. One of the leading explanations for pathways to spontaneous preterm birth is an increase in inflammation at the maternal–fetal interface, leading to early initiation of parturition pathways (Challis et al. 2009). Oxidative stress is tightly linked to inflammation, and could be the origin of inflammation in this pathway. Additionally, recent evidence suggests that oxidative damage to the membranes surrounding the fetus could result in preterm membrane rupture—a subtype of preterm birth that we included in our “spontaneous” category (Menon 2014). This mediation analysis provides a quantitative assertion of this plausible mechanism.

In addition to our substantive findings, we quantitatively compared mediation analyses using average versus repeated measures of the exposure and mediator, as well as the effect of allowing for an exposure–mediator interaction. Fully using repeated measures of exposure and mediator, rather than condensing them to averages, resulted in narrower confidence intervals for indirect effect estimates for models both with and without interaction. This clearly illustrates the improved precision of these advanced mediation models. Furthermore, longitudinal analysis is a more appropriate approach when there is temporal variation in exposure, autocorrelation in the repeated measures, and potential time-varying confounding. Thus, to maintain power and address these issues, longitudinal models should be considered by researchers who have repeated measures of exposure and mediator variables available.

Allowing for an interaction term between exposure and mediator increased the observed percent mediated for both average and repeated measures models. This finding suggests that a biologic interaction may exist in these relationships. Specifically, elevated exposure to phthalates in combination with higher levels of oxidative stress may result in a greater than cumulative increase in risk of spontaneous preterm birth. This has important connotations for future research in environmental impacts on pregnancy, because many contaminants found in pregnant mothers have demonstrated capacity to cause oxidative stress. Examining exposure to mixtures of oxidative stress inducing compounds in relation to preterm birth will be an important next step in this line of research. Notably, although we observed the largest estimated proportion mediated in the model with interaction, this may not be the case for all analyses. Other investigators using a causal framework for mediation analysis should test the inclusion of an exposure–mediator interaction using the models presented here, and should keep these in the model only if they have substantial impact (i.e., show a large change in the estimated proportion mediated), have biologic plausibility, and meet the assumptions described above.

Despite the fact that we noticed a larger estimated proportion of mediated effects and narrower confidence intervals under Method 4, we do not know the truth in this or any given data set and thus cannot claim Method 4 is the best. However, because Method 4 is the most general framework that most fully utilizes the longitudinal information in the data and allows for potential exposure by mediator interaction, an investigator can start with this approach. The advantage of starting with a more general model is that if the interaction has any impact, this approach will capture that situation, and in absence of interaction, the interaction coefficient will be close to null and can be removed.

There are several limitations to our study and the present mediation analysis. In regard to our study design, we measured circulating biomarkers of phthalate exposure and oxidative stress, despite the fact that measures at the maternal–fetal interface may be more biologically relevant. However, markers in urine may be indicative at least in part of activity in the uterine compartment, and collection of urine is much more feasible (i.e., less invasive) than tissue or fluid samples from the uterus during pregnancy. A second limitation is that 8-isoprostane is not a direct measure of reactive oxygen species production but only a proxy. Although we attempted to examine the phthalate–preterm birth mediation by oxidative stress, in reality we only examined the mediation of the relationship by 8-isoprostane, or the mediation by oxidative stress detected by 8-isoprostane in urine. Other factors may influence the relationship between phthalate exposure and 8-isoprostane levels, making 8-isoprostane incompletely representative of the oxidative stress that phthalates produce. Thus the mediation by total oxidative stress may be underestimated in this analysis.

In addition to these study design limitations, the mediation analysis within the counterfactual framework makes strong assumptions for “no unmeasured confounding” of the outcome–exposure, outcome–mediator, and mediator–exposure relationships as described in the Methods section. Although we were able to examine a large number of potential confounders in this analysis, there is always the possibility of unmeasured confounding. One example could include changes in diet, which have been linked to phthalate exposure biomarkers, oxidative stress, and prematurity. However, associations between this potential confounder and preterm birth are questionable, and 8-isoprostane is known for being less sensitive to diet compared to other oxidative stress biomarkers (Milne et al. 2005). Sensitivity analyses can in some instances be applied to theoretically observe how violations of these “no unmeasured confounding” assumptions would affect the mediation results and are described in detail in the literature (VanderWeele and Arah 2011) but are not applied here.

Finally, a major assumption in this analysis is that phthalates cause oxidative stress (i.e., temporal ordering). If oxidative stress causes an increase in urinary phthalate metabolite excretion, or if the connection between the two is not causal but instead a result of unmeasured confounding, then the interpretation of these results would be different. However, based on animal and cellular studies, there is moderate evidence that phthalate exposure during pregnancy causes an increase in reactive oxygen species release which can be measured by urinary excretion of 8-isoprostane (Rusyn et al. 2001; Tetz et al. 2013).

Despite these limitations and assumptions our study has many strengths, namely the ability to examine these associations in a case-control population with a large number of subjects and repeatedly measured biomarkers of both exposure and mediator. It is also the first analysis, to our knowledge, that attempts to identify through an epidemiologic study the mediators of relationships between an environmental contaminant exposure and preterm birth. A number of studies examine relationships between environmental chemicals and prematurity, but are limited by the inability to establish causality. Although mediation analysis still does not concretely establish a causal pathway, it provides an additional step that none of these previous studies have been able to take. These methods may be particularly useful for future studies in this realm, but also for the vast number of epidemiologic studies attempting to identify causal pathways using molecular biomarkers of intermediate effects.

In conclusion, our methodological approach of gradually building more complex mediation models incorporating exposure–mediator interactions and repeated measures of the exposure and mediator illustrate the utility of the longitudinal study design and application of these more sophisticated analytical approaches. Additionally, conditional on the underlying assumptions, these findings provide causal evidence for mediation of the previously observed associations between phthalate exposure and preterm birth by oxidative stress.

## Supplemental Material

(514 KB) PDF
