# Supplementary material for: Mediation of the Relationship between Maternal Phthalate Exposure and Preterm Birth by Oxidative Stress with Repeated Measurements across Pregnancy
Source: Environ Health Perspect. 2016 Jun 28;125(3):488–94. doi: 10.1289/EHP282 (PMC5332184; doi:10.1289/EHP282)
Supplement: (514 KB) PDF [file EHP282.s001.acco.pdf]

Environ Health Perspect
DOI: 10.1289/EHP282

Note to readers with disabilities: *EHP* strives to ensure that all journal content is accessible to all readers. However, some figures and Supplemental Material published in *EHP* articles may not conform to 508 standards due to the complexity of the information being presented. If you need assistance accessing journal content, please contact ehp508@niehs.nih.gov. Our staff will work with you to assess and meet your accessibility needs within 3 working days.

Supplemental Material

Mediation of the Relationship between Maternal Phthalate Exposure and Preterm Birth by Oxidative Stress with Repeated Measurements across Pregnancy

Kelly K. Ferguson, Yin-Hsiu Chen, Tyler J. VanderWeele, Thomas F. McElrath, John D. Meeker, and Bhramar Mukherjee

Table of Contents

Table S1. β coefficients and (SE) from adjusted models used in mediation analysis Method 1: Counterfactual approach utilizing exposure and mediator averages (Models 1-2).

Table S2. β coefficients and (SE) from adjusted models used in mediation analysis Method 2: Counterfactual approach utilizing exposure and mediator averages with interaction (Models 3-4).

Table S3. β coefficients and (SE) from adjusted models used in mediation analysis Method 3: Longitudinal approach utilizing repeated measures of exposure and mediator (Models 5-6).

Table S4. β coefficients and (SE) from adjusted models used in mediation analysis Method 4: Longitudinal approach utilizing repeated measures of exposure and mediator with interaction (Models 7-8).

Table S5. Comparison of effect estimates for mediation Models 7-8 under Method 4 using three different approaches for imputation: 1) Complete case analysis; 2) Imputation with individual average; and 3) Imputation with population average).

Section S1. Statistical models, methods, and R code for mediation of the relationship between maternal phthalate exposure and preterm birth by oxidative stress with repeated measurements across pregnancy.

Table S1. β coefficients and (SE) from adjusted models used in mediation analysis Method 1: Counterfactual approach utilizing exposure and mediator averages (Models 1-2).

All preterm	Model 1 Phthalate metabolite		Model 1 8-Isoprostane		Model 2 Phthalate metabolite	
	β (SE)	p	β (SE)	p	β (SE)	p
MEHP	0.26 (0.13)	0.04	0.68 (0.19)	<0.01	0.09 (0.04)	0.03
MECPP	0.26 (0.12)	0.03	0.66 (0.19)	<0.01	0.17 (0.04)	<0.01
∑DEHP	0.21 (0.13)	0.11	0.68 (0.19)	<0.01	0.15 (0.04)	<0.01
MBP	0.17 (0.14)	0.21	0.69 (0.19)	<0.01	0.16 (0.05)	<0.01
**Spontaneous preterm**	**Model 1 Phthalate metabolite**		**Model 1 8-Isoprostane**		**Model 2 Phthalate metabolite**	
	β (SE)	p	β (SE)	p	β (SE)	p
MEHP	0.48 (0.19)	0.01	1.59 (0.35)	<0.01	0.08 (0.04)	0.06
MECPP	0.27 (0.18)	0.13	1.55 (0.35)	<0.01	0.18 (0.04)	<0.01
∑DEHP	0.34 (0.19)	0.08	1.56 (0.35)	<0.01	0.15 (0.05)	<0.01
MBzP	0.29 (0.21)	0.18	1.53 (0.35)	<0.01	0.09 (0.05)	0.08
MBP	0.30 (0.17)	0.08	1.55 (0.35)	<0.01	0.16 (0.06)	0.01
MiBP	0.27 (0.23)	0.24	1.58 (0.35)	<0.01	0.11 (0.06)	0.04
MEP	0.20 (0.19)	0.28	1.57 (0.35)	<0.01	0.10 (0.05)	0.03
MCPP	0.25 (0.18)	0.17	1.55 (0.35)	<0.01	0.14 (0.05)	<0.01

SE, standard error. [a]Model 1: Preterm birth regressed on average 8-isoprostane and average phthalate metabolite. Model 2: Average 8-isoprostane average regressed on phthalate metabolite average (weighted). All models adjusted for average urinary specific gravity, maternal age, race/ethnicity, education level, health insurance provider, and pre-pregnancy body mass index. Models were built for overall preterm birth (N=461) as well as spontaneous preterm births alone (N=390).

Table S2. β coefficients and (SE) from adjusted models used in mediation analysis Method 2: Counterfactual approach utilizing exposure and mediator averages with interaction (Models 3-4).

All preterm	Model 3 Phthalate metabolite		Model 3 8-Isoprostane		Model 3 Interaction		Model 4 Phthalate metabolite	
	β (SE)	p	β (SE)	p	β (SE)	p	β (SE)	p
MEHP	0.25 (0.13)	0.06	0.77 (0.20)	<0.01	0.18 (0.13)	0.15	0.09 (0.04)	0.03
MECPP	0.26 (0.12)	0.03	0.66 (0.19)	<0.01	0.00 (0.13)	0.99	0.17 (0.04)	<0.01
∑DEHP	0.21 (0.13)	0.11	0.71 (0.19)	<0.01	0.07 (0.13)	0.56	0.15 (0.04)	<0.01
MBP	0.17 (0.14)	0.21	0.68 (0.19)	<0.01	-0.01 (0.13)	0.93	0.16 (0.05)	<0.01
**Spontaneous preterm**	**Model 3 Phthalate metabolite**		**Model 3 8-Isoprostane**		**Model 3 Interaction**		**Model 4 Phthalate metabolite**	
	β (SE)	p	β (SE)	p	β (SE)	p	β (SE)	p
MEHP	0.47 (0.20)	0.02	1.59 (0.35)	<0.01	0.03 (0.25)	0.90	0.08 (0.04)	0.06
MECPP	0.34 (0.19)	0.08	1.56 (0.36)	<0.01	-0.24 (0.25)	0.34	0.18 (0.04)	<0.01
∑DEHP	0.37 (0.20)	0.06	1.58 (0.36)	<0.01	-0.14 (0.25)	0.58	0.15 (0.05)	<0.01
MBzP	0.32 (0.22)	0.15	1.56 (0.35)	<0.01	-0.15 (0.25)	0.54	0.09 (0.05)	0.08
MBP	0.42 (0.20)	0.03	1.58 (0.35)	<0.01	-0.47 (0.28)	0.09	0.16 (0.06)	<0.01
MiBP	0.29 (0.23)	0.21	1.58 (0.35)	<0.01	-0.12 (0.23)	0.61	0.11 (0.06)	0.04
MEP	0.22 (0.20)	0.26	1.57 (0.35)	<0.01	-0.08 (0.26)	0.75	0.10 (0.05)	0.03
MCPP	0.32 (0.19)	0.09	1.60 (0.36)	<0.01	-0.24 (0.23)	0.30	0.14 (0.05)	<0.01

SE, standard error. [a]Model 3: Preterm birth regressed on average 8-isoprostane, average phthalate metabolite, and their interaction term. Model 4: Average 8-isoprostane average regressed on phthalate metabolite average (weighted). All models adjusted for average urinary specific gravity, maternal age, race/ethnicity, education level, health insurance provider, and pre-pregnancy body mass index. Models were built for overall preterm birth (N=461) as well as spontaneous preterm births alone (N=390).

Table S3. β coefficients and (SE) from adjusted models used in mediation analysis Method 3: Longitudinal approach utilizing repeated measures of exposure and mediator (Models 5-6).

All preterm	Model 5 Phthalate metabolite		Model 5 8-Isoprostane		Model 6 Phthalate metabolite (t=1)		Model 6 Phthalate metabolite (t=2)		Model 6 Phthalate metabolite (t=3)	
	β (SE)	p	β (SE)	p	β (SE)	p	β (SE)	p	β (SE)	p
MEHP	0.09 (0.04)	0.04	0.23 (0.06)	<0.01	0.07 (0.03)	0.04	0.08 (0.04)	0.06	0.08 (0.04)	0.06
MECPP	0.09 (0.04)	0.03	0.22 (0.06)	<0.01	0.14 (0.03)	<0.01	0.12 (0.04)	<0.01	0.19 (0.04)	<0.01
∑DEHP	0.07 (0.04)	0.11	0.23 (0.06)	<0.01	0.11 (0.03)	<0.01	0.09 (0.04)	0.04	0.17 (0.04)	<0.01
MBP	0.06 (0.05)	0.21	0.23 (0.06)	<0.01	0.15 (0.05)	<0.01	0.09 (0.06)	0.12	0.15 (0.05)	0.01
**Spontaneous preterm**	**Model 5 Phthalate metabolite**		**Model 5 8-Isoprostane**		**Model 6 Phthalate metabolite (t=1)**		**Model 6 Phthalate metabolite (t=2)**		**Model 6 Phthalate metabolite (t=3)**	
	β (SE)	p	β (SE)	p	β (SE)	p	β (SE)	p	β (SE)	p
MEHP	0.16 (0.06)	0.01	0.53 (0.12)	<0.01	0.07 (0.04)	0.06	0.08 (0.05)	0.12	0.07 (0.05)	0.13
MECPP	0.09 (0.06)	0.13	0.52 (0.12)	<0.01	0.15 (0.04)	<0.01	0.12 (0.05)	0.01	0.20 (0.05)	<0.01
∑DEHP	0.11 (0.06)	0.08	0.52 (0.12)	<0.01	0.12 (0.04)	<0.01	0.09 (0.05)	0.07	0.18 (0.05)	<0.01
MBzP	0.09 (0.07)	0.18	0.51 (0.11)	<0.01	0.16 (0.05)	<0.01	0.04 (0.06)	0.44	0.08 (0.06)	0.14
MBP	0.10 (0.06)	0.08	0.51 (0.12)	<0.01	0.15 (0.05)	<0.01	0.08 (0.06)	0.23	0.15 (0.06)	0.01
MiBP	0.09 (0.08)	0.24	0.52 (0.11)	<0.01	0.15 (0.05)	0.01	0.05 (0.06)	0.42	0.13 (0.06)	0.03
MEP	0.07 (0.06)	0.28	0.52 (0.12)	<0.01	0.15 (0.04)	<0.01	0.09 (0.05)	0.08	0.10 (0.05)	0.06
MCPP	0.08 (0.06)	0.17	0.51 (0.12)	<0.01	0.16 (0.04)	<0.01	0.08 (0.05)	0.15	0.13 (0.05)	0.01

SE, standard error. [a]Model 5: Preterm birth regressed on cumulative 8-isoprostane and cumulative phthalate metabolite in the same model. Model 6: Phthalate metabolite at visit t regressed on average 8-isoprostane up to visit t (weighted). All models adjusted for average urinary specific gravity, maternal age, race/ethnicity, education level, health insurance provider, and pre-pregnancy body mass index. Models were built for overall preterm birth (N=461) as well as spontaneous preterm births alone (N=390).

Table S4. β coefficients and (SE) from adjusted models used in mediation analysis Method 4: Longitudinal approach utilizing repeated measures of exposure and mediator with interaction (Models 7-8).

All preterm	Model 7 Phthalate metabolite		Model 7 8-Isoprostane		Model 7 Interaction		Model 8 Phthalate metabolite (t=1)		Model 8 Phthalate metabolite (t=2)		Model 8 Phthalate metabolite (t=3)	
	β (SE)	p	β (SE)	p	β (SE)	p	β (SE)	p	β (SE)	p	β (SE)	p
MEHP	0.07 (0.04)	0.10	0.26 (0.07)	<0.01	0.02 (0.01)	0.15	0.07 (0.03)	0.01	0.13 (0.03)	<0.01	0.02 (0.01)	0.01
MECPP	0.09 (0.04)	0.04	0.22 (0.07)	<0.01	0.00 (0.01)	0.99	0.11 (0.03)	<0.01	0.15 (0.03)	<0.01	0.00 (0.01)	<0.01
∑DEHP	0.07 (0.04)	0.14	0.24 (0.07)	<0.01	0.01 (0.01)	0.56	0.09 (0.03)	<0.01	0.13 (0.03)	<0.01	0.01 (0.01)	<0.01
MBP	0.06 (0.05)	0.21	0.23 (0.06)	<0.01	0.00 (0.01)	0.93	0.18 (0.04)	<0.01	0.15 (0.04)	<0.01	0.00 (0.01)	<0.01
**Spont. preterm**	**Model 7 Phthalate metabolite**		**Model 7 8-Isoprostane**		**Model 7 Interaction**		**Model 8 Phthalate metabolite (t=1)**		**Model 8 Phthalate metabolite (t=2)**		**Model 8 Phthalate metabolite (t=3)**	
	β (SE)	p	β (SE)	p	β (SE)	p	β (SE)	p	β (SE)	p	β (SE)	p
MEHP	0.16 (0.07)	0.03	0.53 (0.12)	<0.01	0.00 (0.03)	0.90	0.08 (0.03)	0.01	0.12 (0.04)	<0.01	0.08 (0.04)	0.04
MECPP	0.12 (0.07)	0.07	0.52 (0.12)	<0.01	-0.03 (0.03)	0.34	0.13 (0.03)	<0.01	0.14 (0.04)	<0.01	0.18 (0.04)	<0.01
∑DEHP	0.13 (0.07)	0.07	0.52 (0.12)	<0.01	-0.02 (0.03)	0.58	0.11 (0.03)	<0.01	0.12 (0.04)	<0.01	0.16 (0.04)	<0.01
MBzP	0.11 (0.08)	0.14	0.52 (0.12)	<0.01	-0.02 (0.03)	0.54	0.21 (0.04)	<0.01	0.17 (0.05)	<0.01	0.16 (0.05)	<0.01
MBP	0.16 (0.07)	0.02	0.52 (0.12)	<0.01	-0.05 (0.03)	0.09	0.16 (0.04)	<0.01	0.11 (0.04)	0.01	0.14 (0.04)	<0.01
MiBP	0.10 (0.08)	0.20	0.53 (0.12)	<0.01	-0.01 (0.03)	0.61	0.27 (0.05)	<0.01	0.16 (0.05)	<0.01	0.19 (0.05)	<0.01
MEP	0.08 (0.07)	0.27	0.52 (0.12)	<0.01	-0.01 (0.03)	0.75	0.18 (0.04)	<0.01	0.13 (0.04)	<0.01	0.14 (0.04)	<0.01
MCPP	0.12 (0.07)	0.08	0.52 (0.12)	<0.01	-0.03 (0.03)	0.30	0.15 (0.03)	<0.01	0.10 (0.04)	0.01	0.13 (0.04)	<0.01

Spont, spontaneous; SE, standard error. [a]Model 7: Preterm birth regressed on cumulative 8-isoprostane, cumulative phthalate metabolite, and their cross-product interaction in the same model. Model 8: Phthalate metabolite at visit t regressed on average 8-isoprostane up to visit t (weighted) with the regression coefficients for t = 1, 2, 3 jointly estimated. All models adjusted for average urinary specific gravity, maternal age, race/ethnicity, education level, health insurance provider, and pre-pregnancy body mass index. Models were built for overall preterm birth (N=461) as well as spontaneous preterm births alone (N=390).

Table S5. Comparison of effect estimates for mediation Models 7-8 under Method 4 using three different approaches for imputation: 1) Complete case analysis; 2) Imputation with individual average; and 3) Imputation with population average.

	1) Complete case analysis				2) Imputation with individual average				3) Imputation with population average			
All preterm	NDE	NIE	Total effect	Percent mediated	NDE	NIE	Total effect	Percent mediated	NDE	NIE	Total effect	Percent mediated
MEHP	0.244	0.068	0.311	22%	0.298	0.077	0.374	20%	0.285	0.081	0.365	22%
MECPP	0.237	0.102	0.339	30%	0.264	0.094	0.358	26%	0.264	0.093	0.357	26%
∑DEHP	0.220	0.088	0.308	28%	0.224	0.086	0.310	28%	0.219	0.086	0.305	28%
MBP	0.123	0.101	0.224	45%	0.168	0.112	0.280	40%	0.168	0.103	0.271	38%
	1) Complete case analysis				2) Imputation with individual average				3) Imputation with population average			
Spont. preterm	NDE	NIE	Total effect	Percent mediated	NDE	NIE	Total effect	Percent mediated	NDE	NIE	Total effect	Percent mediated
MEHP	0.332	0.086	0.419	21%	0.501	0.147	0.649	23%	0.497	0.154	0.650	24%
MECPP	0.103	0.160	0.264	61%	0.160	0.235	0.395	60%	0.194	0.227	0.421	54%
∑DEHP	0.159	0.128	0.287	45%	0.268	0.202	0.469	43%	0.287	0.198	0.486	41%
MBzP	0.050	0.223	0.272	82%	0.220	0.282	0.502	56%	0.239	0.260	0.499	52%
MBP	0.080	0.157	0.237	66%	0.049	0.212	0.212	81%	0.116	0.216	0.332	65%
MiBP	0.085	0.241	0.326	74%	0.207	0.327	0.534	61%	0.222	0.286	0.508	56%
MEP	0.200	0.182	0.382	48%	0.163	0.238	0.401	59%	0.175	0.253	0.428	59%
MCPP	0.097	0.127	0.224	57%	0.141	0.199	0.340	59%	0.179	0.208	0.388	54%

Spont, spontaneous; NDE, natural direct effect; NIE, natural indirect effect.

Mediation of the Relationship between Maternal Phthalate Exposure and Preterm Birth by Oxidative Stress with Repeated Measurements across Pregnancy

Kelly K. Ferguson, Yin-Hsiu Chen, Tyler J. VanderWeele, Thomas F. McElrath, John D. Meeker, and Bhramar Mukherjee

Supplemental Material

Section S1 Steps for Mediation Analysis with Longitudinal Exposure and Mediator with a Binary Outcome

Notation

- N: Number of subjects
- $A_i(t)$: Centered log of urinary phthalate MEHP (continuous exposure) at visit t ($t = 1, 2, 3$) for subject i
- $M_i(t)$: Centered log of urinary 8-isoprostane (continuous mediator) at visit t ($t = 1, 2, 3$) for subject i
- Y_i: Preterm birth status (binary outcome) for subject i
- V_i: Vector of time-invariant covariates including maternal age, race/ethnicity, education level, health-insurance provider, pre-pregancy BMI
- $L_i(t)$: Potential time-varying confounders or covariates: In our dataset we have only one time-varying variable specific gravity measured at visit t ($t = 1, 2, 3$) for subject i, it is needed for adjustment when phthalate levels are used as predictors in a model but not a confounder.

Note:

- The exposure and mediator are standardized by the standard error after logarithm transformation for comparability between different exposures and better interpretability.

Method 1 - Counterfactual Approach with Average Exposure and Average Mediator without interaction (Vanderweele and Vansteelandt, 2010)

Under assumptions (A1)-(A4) described above, the following model can be used to estimate direct and indirect effects:

Model 1 (Logistic Regression):

$$\text{logit}[P(Y_i = 1|A_i(t), M_i(t), V_i, L_i(t))] = \beta_{y0} + \beta_{ya}\bar{A}_i + \beta_{ym}\bar{M}_i + \beta_{yv}V_i + \beta_{yl}\bar{L}_i$$

where $\bar{A}_i = \sum_{t=1}^{3} A_i(t)/3$, $\bar{M}_i = \sum_{t=1}^{3} M_i(t)/3$, and $\bar{L}_i = \sum_{t=1}^{3} L_i(t)/3$

Model 2 (Linear Regression):

$$E[M_i|A_i(t), V_i, L_i(t)] = \beta_{m0} + \beta_{ma}\bar{A}_i + \beta_{mv}V_i + \beta_{ml}\bar{L}_i$$

where $\bar{A}_i = \sum_{t=1}^{3} A_i(t)/3$ and $\bar{L}_i = \sum_{t=1}^{3} L_i(t)/3$

with IPW for case-control sampling

Expression of Mediation Effects:

Let σ^2 denote the variance of the error term in Model 2. One unit increase in the average exposure level (centered) from $\bar{a}^* = -1$ to $\bar{a} = 0$ corresponds to

Controlled Direct Effect (logarithm scale) = β_{ya}
Natural Direct Effect (logarithm scale) = β_{ya}
Natural Indirect Effect (logarithm scale) = $\beta_{ym}\beta_{ma}$
Proportion Mediated = $\frac{\beta_{ym}\beta_{ma}}{\beta_{ym}\beta_{ma}+\beta_{ya}}$

Note:

- The standard errors of natural indirect effect are estimated via bootstrapping.

Method 2 - Counterfactual Approach with Average Exposure and Average Mediator and their interaction (Vanderweele and Vansteelandt, 2010)

Under assumptions (A1)-(A4) described above, the following model can be used to estimate direct and indirect effects:

Model 3 (Logistic Regression):

$$\text{logit}[P(Y_i = 1|A_i(t), M_i(t), V_i, L_i(t))] = \beta_{y0} + \beta_{ya}\bar{A}_i + \beta_{ym}\bar{M}_i + \beta_{yi}\bar{A}_i\bar{M}_i + \beta_{yv}V_i + \beta_{yl}\bar{L}_i$$

where $\bar{A}_i = \sum_{t=1}^{3} A_i(t)/3$, $\bar{M}_i = \sum_{t=1}^{3} M_i(t)/3$, and $\bar{L}_i = \sum_{t=1}^{3} L_i(t)/3$

Model 4 (Linear Regression):

$$E[M_i|A_i(t), V_i, L_i(t)] = \beta_{m0} + \beta_{ma}\bar{A}_i + \beta_{mv}V_i + \beta_{ml}\bar{L}_i$$

where $\bar{A}_i = \sum_{t=1}^{3} A_i(t)/3$ and $\bar{L}_i = \sum_{t=1}^{3} L_i(t)/3$

with IPW for case-control sampling

Expression of Mediation Effects:

Let σ^2 denote the variance of the error term in Model 4. One unit increase in the average exposure level (centered) from $\bar{a}^* = -1$ to $\bar{a} = 0$, when the average mediator (centered) is set at $\bar{m} = 0$, continuous covariates are set at their average levels, and categorical covariates are set at 0 (reference group), corresponds to

Controlled Direct Effect (logarithm scale) = $(\beta_{ya} + \beta_{yi}\bar{m})$
Natural Direct Effect (logarithm scale) = $[\beta_{ya} + \beta_{yi}(\beta_{m0} - \beta_{ma} + \beta_{mv}\bar{v} + \beta_{ml}\bar{l} + \beta_{ym}\sigma^2)] - 0.5\beta_{yi}^2\sigma^2$
Natural Indirect Effect (logarithm scale) = $\beta_{ym}\beta_{ma}$
Proportion Mediated = $\frac{\text{Indirect Effect}}{\text{Direct Effect + Indirect Effect}}$

Note:

- The standard errors of controlled direct effect, natural direct effect, and natural indirect effect are estimated via bootstrapping.

Method 3 - Counterfactual Approach with Longitudinal Exposure and Longitudinal Mediator without Interaction (VanderWeele and Tchetgen Tchetgen, 2014)

Mediation Ordering in Time

$$\begin{array}{ccccccccccccc} V_i & \rightarrow & A_i(1) & \rightarrow & M_i(1) & \rightarrow & A_i(2) & \rightarrow & M_i(2) & \rightarrow & A_i(3) & \rightarrow & M_i(3) & \rightarrow & Y_i \\ & & \uparrow & & & & \uparrow & & & & \uparrow & & & & \\ & & L_i(1) & & & & L_i(2) & & & & L_i(3) & & & & \end{array}$$

Marginal Structural Models (MSMs)

Under a longitudinal version of assumptions (A1)-(A4) (see VanderWeele and Tchetgen Tchetgen, 2014), we can use the following models to estimate direct and indirect effects.

Model 5 - Logistic regression model for $\boldsymbol{Y}$:

$$\text{logit}[P(Y_i = 1|A_i(t), M_i(t), V_i, L_i(t))] = \beta_{y0} + \beta_{ya} cum_i(\bar{a}) + \beta_{ym} cum_i(\bar{m}) + \beta_{yv} V_i + \beta_{yl} \bar{L}_i$$

where $cum_i(\bar{a}) = \sum_{t=1}^{3} A_i(t)$, $cum_i(\bar{m}) = \sum_{t=1}^{3} M_i(t)$, and $\bar{L}_i = \sum_{t=1}^{3} L_i(t)/3$

Remark: In the absence of time-varying confounding, the conditional models above can be interpreted as structural models. If there exists time-varying confounder(s) $C_i(t)$, an extra term $\beta_{yc} cum_i(\bar{c})$ where $cum_i(\bar{c}) = \sum_{t=1}^{3} C_i(t)$ is added to the logistic regression model. The consistent coefficient estimates can be obtained by weighted regression with stabilized IPW:

$$\prod_{t=1}^{3} \frac{f[M_i(t)|A_i(t), M_i(t-1), V_i, L_i(t)]}{f[M_i(t)|A_i(t), M_i(t-1), C_i(t-1), V_i, L_i(t)]} \frac{f[A_i(t)|A_i(t-1), M_i(t-1), V_i, L_i(t)]}{f[A_i(t)|A_i(t-1), M_i(t-1), C_i(t-1), V_i, L_i(t)]}$$

where $f(\cdot)$ is the density function

Model 6 - Three linear regression models for $\boldsymbol{M_i(t)}$

$$E[M_i(t)|A_i(t), V_i, L_i(t)] = \beta_{m0}(t) + \beta_{ma}(t) avg_i(\bar{a}(t)) + \beta_{mv}(t) V_i + \beta_{ml}(t) L_i(t)$$

where $avg_i(\bar{a}(t)) = \sum_{s=1}^{t} A_i(s)/t$ for $t = 1, 2, 3$

with IPW for case-control sampling.

If there exists time-varying confounder(s) $C_i(t)$, an extra term $\beta_{mc}(t) C_i(t)$ is added to each of three regression models. The consistent coefficient estimates can be obatined by weighted regression with stabilized IPW:

$$\prod_{s=1}^{t} \frac{f[A_i(s)|A_i(s-1), M_i(s-1), V_i, L_i(s)]}{f[A_i(s)|A_i(s-1), M_i(s-1), C_i(s-1), V_i, L_i(s)]} \qquad \text{for } t = 1, 2, 3$$

in additional to the IPW for case-control sampling.

Expression of Mediation Effects:

Scenario: One unit increase in the exposure level at each of the $t = 1, 2, 3$
(i.e. $a_1 = a_1^* + 1, a_2 = a_2^* + 1, a_3 = a_3^* + 1$)

$$cum_i(\bar{a}) = cum_i(\bar{a}^*) + 3$$

$$avg_i(\bar{a}(1)) = avg_i(\bar{a}^*(1)) + 1$$

$$avg_i(\bar{a}(2)) = avg_i(\bar{a}^*(2)) + 1$$

$$avg_i(\bar{a}(3)) = avg_i(\bar{a}^*(3)) + 1$$

Natural Direct Effect (logarithm scale) = $3\beta_{ya}$
Natural Indirect Effect (logarithm scale) = $\beta_{ym}[\beta_{ma}(1) + \beta_{ma}(2) + \beta_{ma}(3)]$

The proportion mediated (in logarithm scale) can be expressed as:

$$\frac{\beta_{ym}[\beta_{ma}(1)+\beta_{ma}(2)+\beta_{ma}(3)]}{3\beta_{ya}+\beta_{ym}[\beta_{ma}(1)+\beta_{ma}(2)+\beta_{ma}(3)]}$$

Note:

- The standard errors of natural direct effect and natural indirect effect are estimated via bootstrapping.

Method 4 - Counterfactual Approach with Longitudinal Exposure and Longitudinal Mediator with Interaction (VanderWeele and Tchetgen Tchetgen, 2014)

Marginal Structural Models (MSMs)

Under a longitudinal version of assumptions (A1)-(A4) (see VanderWeele and Tchetgen Tchetgen, 2014), we can use the following models to estimate direct and indirect effects.

Model 7 - Logistic regression model for $\boldsymbol{Y}$:

$$\begin{aligned}\text{logit}[P(Y_i=1|A_i(t),M_i(t),V_i,L_i(t))] =& \beta_{y0}+\beta_{ya}cum_i(\bar{a})+\beta_{ym}cum_i(\bar{m})\\ &+\beta_{yi}cum_i(\bar{a})cum_i(\bar{m})\beta_{yv}+V_i+\beta_{yl}\bar{L}_i\end{aligned}$$

where $cum_i(\bar{a})=\sum_{t=1}^{3}A_i(t)$, $cum_i(\bar{m})=\sum_{t=1}^{3}M_i(t)$, and $\bar{L}_i=\sum_{t=1}^{3}L_i(t)/3$

If there exists time-varying confounder(s) $C_i(t)$, consistent coefficient estimates can be obtained by incorporating the stabilized IPW given in model 5.

Model 8 - Three linear regression models for $\boldsymbol{M_i(t)}$ with correlated residuals:

$$E[M_i(t)|A_i(t),V_i,L_i(t)]=\beta_{m0}(t)+\beta_{ma}(t)avg_i(\bar{a}(t))+\beta_{mv}(t)V_i+\beta_{ml}(t)L_i(t)$$

where $avg_i(\bar{a}(t))=\sum_{s=1}^{t}A_i(s)/t$ for $t=1,2,3$ and the residuals from the three models $(\epsilon_i(1),\epsilon_i(2),\epsilon_i(3))^T$ follows a zero-mean multivariate Gaussian distribution with common variance and unstructured correlation. The linear regression models are adjusted for case-control sampling with IPW.

If there exists time-varying confounder(s) $C_i(t)$, consistent coefficient estimates can be obtained by incorporating the stabilized IPW given in model 6 on the top of IPW for case-control sampling.

Expression of Mediation Effects

Scenario: One unit increase in the exposure level at each of the $t=1,2,3$ from -1 to 0 (i.e. $a_1=a_2=a_3=0, a_1^*=a_2^*=a_3^*=-1$)

$$cum_i(\bar{a})=0$$

$$cum_i(\bar{a}^*)=-3$$

$$avg_i(\bar{a}(1))=avg_i(\bar{a}(2))=avg_i(\bar{a}(3))=0$$

$$avg_i(\bar{a}^*(1))=avg_i(\bar{a}^*(2))=avg_i(\bar{a}^*(3))=-1$$

Natural Direct Effect (logarithm scale) =

$$3\Big\{\beta_{ya}+\{\beta_{yi}\textstyle\sum_{t=1}^{3}[\beta_{m0}(t)-\beta_{ma}(t)+\beta_{mv}(t)\bar{v}+\beta_{ml}(t)\bar{l}]+\sigma^2\beta_{ym}\}\Big\}-\frac{9}{2}\sigma^2\beta_{yi}^2$$

Natural Indirect Effect (logarithm scale) = $\beta_{ym}\sum_{t=1}^{3}\beta_{ma}(t)$

The proportion mediated (in logarithm scale) can be expressed as:

$$\frac{\text{Natural Indirect Effect}}{\text{Natural Direct Effect}+\text{Natural Indirect Effect}}$$

where β_{yi} is the coefficient of the interaction term $(cum_i(\bar{a})cum_i(\bar{m}))$ added to Model 6, σ^2 is the variance of $cum_i(\bar{m})$, and $\bar{v}$ is set at mean levels for continous covariates and 0 (reference group) for categorical covariates.

Note:

- The standard errors of natural direct effect and natural indirect effect are estimated via bootstrapping.

Derivations of Effect Expressions in Method 4

Using the notation, definitions, and assumptions of VanderWeele and Tchetgen Tchetgen (2014) we can proceed as follows. The natural direct effect (NDE) and natural indirect effect (NIE) in logarithm scale with exposure changed from $\bar{a}^*$ to $\bar{a}$ are given by

$$\log(OR^{NDE}) = \text{logit}[P(Y_{\bar{a}M_{\bar{a}^*}} = 1)] - \text{logit}[P(Y_{\bar{a}^*M_{\bar{a}^*}} = 1)]$$

$$\log(OR^{NIE}) = \text{logit}[P(Y_{\bar{a}M_{\bar{a}}} = 1)] - \text{logit}[P(Y_{\bar{a}M_{\bar{a}^*}} = 1)].$$

Denote $cum(M) = \sum_{i=1}^{3} M(t)$. From the specifications of three linear regression models for $M(t)$ above, we can obtain that

$$cum(M)|\bar{a}, v, l \sim N(\sum_{t=1}^{3}[\beta_{m0}(t) + \beta_{ma}(t)avg(\bar{a}(t)) + \beta_{mv}(t)v + \beta_{ml}(t)l], \sigma^2).$$

$\sigma^2 = \mathbf{1}^T\mathbf{\Sigma}\mathbf{1}$ where $\mathbf{1} = (1,1,1)^T$ and $\mathbf{\Sigma}$ is the variance-covariance matrix of the error terms from the three regression models (model 8). For convenience, we denote $cum(\bar{m}) = c_m$. It follows that

$$\begin{aligned}
&\text{logit}\{P(Y_{\bar{a}M_{\bar{a}^*}} = 1)\} \\
\approx&\log\{P(Y_{\bar{a}M_{\bar{a}^*}} = 1)\} \\
=&\log\{\int P(Y_{\bar{a}\bar{m}} = 1|\bar{a}, M_{\bar{a}^*} = \bar{m}, v, l)]P(M_{\bar{a}^*} = \bar{m}|\bar{a}^*, v, l)\ d\bar{m}\} \\
\approx&\log\{\int \exp[\beta_{y0} + \beta_{ya}cum(\bar{a}) + \beta_{ym}c_m + \beta_{yi}cum(\bar{a})c_m + \beta_{yv}v + \beta_{yl}l]P(cum(M) = c_m|\bar{a}^*, v, l)\ dc_m\} \\
=&\log\{\exp[\beta_{y0} + \beta_{ya}cum(\bar{a}) + \beta_{yv}v + \beta_{yl}l]\int e^{[\beta_{ym}+\beta_{yi}cum(\bar{a})]c_m}P(cum(M) = c_m|\bar{a}^*, v, l)\ dc_m\} \\
=&\beta_{y0} + \beta_{ya}cum(\bar{a}) + \beta_{yv}v + \beta_{yl}l + \log[E(e^{[\beta_{ym}+\beta_{yi}cum(\bar{a})]cum(M)}|\bar{a}^*, v, l)] \\
=&\beta_{y0} + \beta_{ya}cum(\bar{a}) + \beta_{yv}v + \beta_{yl}l \\
&+ \log\{\exp\{[\beta_{ym} + \beta_{yi}cum(\bar{a})]\sum_{t=1}^{3}[\beta_{m0}(t) + \beta_{ma}(t)avg(\bar{a}^*(t)) + \beta_{mv}(t)v + \beta_{ml}(t)l] \\
&+ \frac{1}{2}\sigma^2[\beta_{ym} + \beta_{yi}cum(\bar{a})]^2\}\} \\
=&\beta_{y0} + \beta_{ya}cum(\bar{a}) + \beta_{yv}v + \beta_{yl}l \\
&+ [\beta_{ym} + \beta_{yi}cum(\bar{a})]\sum_{t=1}^{3}[\beta_{m0}(t) + \beta_{ma}(t)avg(\bar{a}^*(t)) + \beta_{mv}(t)v + \beta_{ml}(t)l] \\
&+ \frac{1}{2}\sigma^2[\beta_{ym} + \beta_{yi}cum(\bar{a})]^2
\end{aligned}$$

Simiarly,

$$
\begin{aligned}
&\text{logit}\{P(Y_{\bar{a}^* M_{\bar{a}^*}} = 1)\} \\
\approx&\beta_{y0} + \beta_{ya} cum(\bar{a}^*) + \beta_{yv} v + \beta_{yl} l \\
&+ [\beta_{ym} + \beta_{yi} cum(\bar{a}^*)] \sum_{t=1}^{3} [\beta_{m0}(t) + \beta_{ma}(t) avg(\bar{a}^*(t)) + \beta_{mv}(t) v + \beta_{ml}(t) l] \\
&+ \frac{1}{2}\sigma^2 [\beta_{ym} + \beta_{yi} cum(\bar{a}^*)]^2
\end{aligned}
$$

$$
\begin{aligned}
&\text{logit}\{P(Y_{\bar{a} M_{\bar{a}}} = 1)\} \\
\approx&\beta_{y0} + \beta_{ya} cum(\bar{a}) + \beta_{yv} v + \beta_{yl} l \\
&+ [\beta_{ym} + \beta_{yi} cum(\bar{a})] \sum_{t=1}^{3} [\beta_{m0}(t) + \beta_{ma}(t) avg(\bar{a}(t)) + \beta_{mv}(t) v + \beta_{ml}(t) l] \\
&+ \frac{1}{2}\sigma^2 [\beta_{ym} + \beta_{yi} cum(\bar{a})]^2
\end{aligned}
$$

Therefore,

$$
\begin{aligned}
\log(OR^{NDE}) &= \text{logit}[P(Y_{\bar{a} M_{\bar{a}^*}} = 1)] - \text{logit}[P(Y_{\bar{a}^* M_{\bar{a}^*}} = 1)] \\
&= \beta_{ya}[cum(\bar{a}) - cum(\bar{a}^*)] \\
&\quad + \beta_{yi}[cum(\bar{a}) - cum(\bar{a}^*)] \sum_{t=1}^{3} [\beta_{m0}(t) + \beta_{ma}(t) avg(\bar{a}^*(t)) + \beta_{mv}(t) v + \beta_{ml}(t) l] \\
&\quad + \frac{1}{2}\sigma^2 \{[\beta_{ym} + \beta_{yi} cum(\bar{a})]^2 - [\beta_{ym} + \beta_{yi} cum(\bar{a}^*)]^2\} \\
&= [cum(\bar{a}) - cum(\bar{a}^*)] \Big\{ \beta_{ya} + \beta_{yi} \sum_{t=1}^{3} [\beta_{m0}(t) + \beta_{ma}(t) avg(\bar{a}^*(t)) + \beta_{mv}(t) v + \beta_{ml}(t) l] \\
&\quad + \sigma^2 \beta_{ym} \Big\} + \frac{1}{2}\sigma^2 \beta_{yi}^2 [cum(\bar{a})^2 - cum(\bar{a}^*)^2]
\end{aligned}
$$

$$
\begin{aligned}
\log(OR^{NIE}) &= \text{logit}[P(Y_{\bar{a} M_{\bar{a}}} = 1)] - \text{logit}[P(Y_{\bar{a} M_{\bar{a}^*}} = 1)] \\
&= \sum_{t=1}^{3} [\beta_{ym} + \beta_{yi} cum(\bar{a})] \beta_{ma}(t) [avg(\bar{a}(t)) - avg(\bar{a^*}(t))]
\end{aligned}
$$

R Code

```
## method 1 & 2
n <- dim(dat)[1]
mod1 <- glm(PRETERM ~ biomarker*log.iso_avg + sg_avg + age + race_cat_new + edu_cat_new
+ insur_new + bmi_cat_new, family = binomial(link = "logit"), data = data.frame(dat))
# for method 1, replace 'biomarker*log.iso_avg' with 'biomarker + log.iso_avg' in the above
formula
mod2 <- lm(log.iso_avg ~ biomarker + sg_avg + age + race_cat_new + edu_cat_new + insur_new
+ bmi_cat_new, data = data.frame(dat), weights = weight)
theta1 <- mod1$coef[2]
sd.theta1 <- summary(mod1)$coef[2, 2]
pval.theta1 <- summary(mod1)$coef[2, 4]
theta2 <- mod1$coef[3]
sd.theta2 <- summary(mod1)$coef[3, 2]
pval.theta2 <- summary(mod1)$coef[3, 4]
theta3 <- mod1$coef[14]
sd.theta3 <- summary(mod1)$coef[14, 2]
```

```
pval.theta3 <- summary(mod1)$coef[14, 4]
beta0 <- mod2$coef[1]
beta1 <- mod2$coef[2]
sd.beta1 <- summary(mod2)$coef[2, 2]
pval.beta1 <- summary(mod2)$coef[2, 4]
beta2 <- mod2$coef[3:12]
method2_spon_coef[k, ] <- as.numeric(c(theta1, sd.theta1, pval.theta1, theta2, sd.theta2,
pval.theta2, theta3, sd.theta3, pval.theta3, beta1, sd.beta1, pval.beta1))
sigma2 <- (summary(mod2)$sigma)^2
a <- mean(dat$biomarker)
a0 <- a - 1
c <- c(median(dat$sg_avg), median(dat.temp$age, na.rm = TRUE), rep(0, 8))
c.all <- matrix(0, 72, 10)
c.all[, 1] <- median(dat$sg_avg)
c.all[, 2] <- median(dat$age, na.rm = TRUE)
c.all[, 3] <- rep(c(0, 1, 0), each = 24)
c.all[, 4] <- rep(c(0, 0, 1), each = 24)
c.all[, 5] <- rep(c(0, 1, 0, 0), each = 6, times = 3)
c.all[, 6] <- rep(c(0, 0, 1, 0), each = 6, times = 3)
c.all[, 7] <- rep(c(0, 0, 0, 1), each = 6, times = 3)
c.all[, 8] <- rep(c(0, 1), each = 3, times = 12)
c.all[, 9] <- rep(c(0, 1, 0), 24)
c.all[, 10] <- rep(c(0, 0, 1), 24)
m <- median(dat$log.iso_avg)
log.or.cde <- as.numeric((theta1 + theta3*m)*(a - a0))
log.or.nde <- as.numeric((theta1 + theta3*(beta0 + beta1*a0 + c.all%*%beta2 + theta2*sigma2))*(a
- a0) + 0.5*theta3^2*sigma2*(a^2 - a0^2))
log.or.nie <- as.numeric((theta2*beta1 + theta3*beta1*a)*(a - a0))
log.or.tot <- log.or.nde + log.or.nie
or.cde <- exp(log.or.cde)
or.nde <- exp(log.or.nde)
or.nie <- exp(log.or.nie)
or.tot <- or.nde*or.nie
pct.med <- log.or.nie / log.or.tot

## bootstrapping to estimate the standard errors
dat <- dat[complete.cases(dat), ]
nboot <- 10000
theta1.boot <- theta2.boot <- theta3.boot <- beta0.boot <- beta1.boot <- sigma2.boot <-
a0.boot <- a.boot <- m.boot <- log.or.cde.boot <- log.or.nie.boot <- rep(0, nboot)
beta2.boot <- mat.or.vec(nboot, 10)
log.or.nde.boot <- mat.or.vec(nboot, 72)

for(i in 1:nboot) {
dat.boot <- dat[sample(1:n, n, replace = TRUE), ]
fac.boot <- sum(dat.boot$weight)/n
mod1.boot <- glm(PRETERM ~ biomarker*log.iso_avg + sg_avg + age + race_cat_new + edu_cat_new
+ insur_new + bmi_cat_new, family = binomial(link = "logit"), data = data.frame(dat.boot))
# for method 1, replace 'biomarker*log.iso_avg' with 'biomarker + log.iso_avg' in the above
formula
mod2.boot <- lm(log.iso_avg ~ biomarker + sg_avg + age + race_cat_new + edu_cat_new + insur_new
+ bmi_cat_new, data = data.frame(dat.boot), weights = weight/fac.boot)
theta1.boot[i] <- mod1.boot$coef[2]
theta2.boot[i] <- mod1.boot$coef[3]
theta3.boot[i] <- mod1.boot$coef[14]
beta0.boot[i] <- mod2.boot$coef[1]
beta1.boot[i] <- mod2.boot$coef[2]
beta2.boot[i, ] <- mod2.boot$coef[3:12]
```

```
sigma2.boot[i] <- (summary(mod2.boot)$sigma)^2
a.boot[i] <- mean(dat.boot$biomarker)
a0.boot[i] <- a.boot[i] - 1
c.all.boot <- matrix(0, 72, 10)
c.all.boot[, 1] <- median(dat.boot$sg_avg)
c.all.boot[, 2] <- median(dat.boot$age)
c.all.boot[, 3:10] <- c.all[, 3:10]
m.boot[i] <- median(dat.boot$log.iso_avg)
log.or.cde.boot[i] <- as.numeric((theta1.boot[i] + theta3.boot[i]*m.boot[i])* (a.boot[i]
- a0.boot[i]))
log.or.nde.boot[i, ] <- as.numeric((theta1.boot[i] + theta3.boot[i]*(beta0.boot[i] + beta1.boot[i]*a
+ beta2.boot[i, ]%*%t(c.all.boot) +theta2.boot[i]*sigma2.boot[i]))*(a.boot[i] - a0.boot[i])
+ 0.5*(theta3.boot[i])^2*(sigma2.boot[i])*((a.boot[i])^2  - (a0.boot[i])^2))
log.or.nie.boot[i] <- as.numeric((theta2.boot[i]*beta1.boot[i] + theta3.boot[i]*beta1.boot[i]*a.boot
 - a0.boot[i]))
summary.tab <- mat.or.vec(72, 32)
colnames(summary.tab) <- c("Race", "Edu", "Insur", "Bmi", "log(CDE)", "log(CDE-lower)",
"log(CDE-upper)", "log(NDE)", "log(NDE-lower)", "log(NDE-upper)", "log(NIE)", "log(NIE-lower)",
"log(NIE-upper)", "log(TT)", "log(TT-lower)", "log(TT-upper)", "Prop", "CDE", "CDE-lower",
"CDE-upper", "NDE", "NDE-lower", "NDE-upper", "NIE", "NIE-lower",
"NIE-upper", "TT", "TT-lower", "TT-upper", "TT2", "Prop-lower", "Prop-Upper")
summary.tab[, 1] <- c.all[, 3] + c.all[, 4]*2
summary.tab[, 2] <- c.all[, 5] + c.all[, 6]*2 + c.all[, 7]*3
summary.tab[, 3] <- c.all[, 8]
summary.tab[, 4] <- c.all[, 9] + c.all[, 10]*2
summary.tab[, 5] <- log.or.cde
summary.tab[, 6] <- log.or.cde.boot[order(log.or.cde.boot)][nboot*0.025]
summary.tab[, 7] <- log.or.cde.boot[order(log.or.cde.boot)][nboot*0.975]
summary.tab[, 8] <- log.or.nde
summary.tab[, 9] <- apply(log.or.nde.boot, 2, function(x) x[order(x)][nboot*0.025])
summary.tab[, 10] <- apply(log.or.nde.boot, 2, function(x) x[order(x)][nboot*0.975])
summary.tab[, 11] <- log.or.nie
summary.tab[, 12] <- log.or.nie.boot[order(log.or.nie.boot)][nboot*0.025]
summary.tab[, 13] <- log.or.nie.boot[order(log.or.nie.boot)][nboot*0.975]
log.or.tot.boot <- log.or.nde.boot + log.or.nie.boot
summary.tab[, 14] <- summary.tab[, 8] + summary.tab[, 11]
summary.tab[, 15] <- apply(log.or.tot.boot, 2, function(x) x[order(x)][nboot*0.025])
summary.tab[, 16] <- apply(log.or.tot.boot, 2, function(x) x[order(x)][nboot*0.975])
summary.tab[, 17] <- summary.tab[, 11] / summary.tab[, 14]
summary.tab[, 18:29] <- exp(summary.tab[, 5:16])
summary.tab[, 30] <- summary.tab[, 21]*summary.tab[, 24] # exp(summary.tab[, 14])
prop.boot <- log.or.nie.boot / log.or.tot.boot
summary.tab[, 31] <- apply(prop.boot, 2, function(x) x[order(x)][nboot*0.025])
summary.tab[, 32] <- apply(prop.boot, 2, function(x) x[order(x)][nboot*0.975])
}

## method 3 & 4: longitudinal analysis
require(nlme)
require(Matrix)
sg_avg <- apply(dat[, c(265, 276, 287)], 1, function(x) mean(x, na.rm = TRUE))
weight <- dat$PRETERM*1.1 + (1 - dat$PRETERM)*2.95

# longitudinal mediation analysis with bootstrap sampling nboot <- 10000
coef.boot <- array(0, dim = c(8, 29, nboot))
coef.tab <- coef.sd.tab <- pval.tab <- matrix(0, 8, 29)
eff.boot <- array(0, dim = c(8, 8, nboot))
sigma2.boot <- matrix(0, 8, nboot)
eff.tab <- matrix(0, 8, 8)
```

```
sg_mean_boot <- array(0, dim = c(8, 3, nboot))
age_mean_boot <- matrix(0, 8, nboot)
colnames(eff.tab) <- c("Direct Effect (without interaction)", "Indirect Effect (without
interaction)", "Prop Effect (without interaction)", "Direct Effect (with interaction)",
"Indirect Effect (with interaction)", "Prop Effect (with interaction)", "Total Effect (without
interaction)", "Total Effect (with interaction)")
dat1 <- dat.temp1[complete.cases(dat1), ]
colnames(dat1)[1:4] <- c(paste("a", 1:3, sep = ""), "aavg")
isosum <- dat1$isoavg*3
asum <- dat1$aavg*3
aavg_2 <- (dat1$a1 + dat1$a2)/2
dat2 <- cbind(dat1, isosum, asum, aavg_2)
dat2$weight <- dat.temp2$weight / sum(dat.temp2$weight) * dim(dat2)[1]
mod.y <- glm(PRETERM ~ asum + isosum + sg_avg + age + race_cat_new+ insur_new + edu_cat_new
+ bmi_cat_new, dat = dat, family = binomial(link = "logit"), control = list(maxit = 10000))
mod.m3 <- lm(iso_3 ~ aavg + sg_3 + age + race_cat_new + insur_new + edu_cat_new + bmi_cat_new,
dat = dat2, weights = weight)
mod.m2 <- lm(iso_2 ~ aavg_2 + sg_2 + age + race_cat_new + insur_new + edu_cat_new + bmi_cat_new,
dat = dat2, weights = weight)
mod.m1 <- lm(iso_1 ~ a1 + sg_1 + age + race_cat_new + insur_new + edu_cat_new + bmi_cat_new,
dat = dat2, weights = weight)
mod.y.int <- glm(PRETERM ~ asum + isosum + asum*isosum + sg_avg + age + race_cat_new + insur_new
+ edu_cat_new + bmi_cat_new, dat = dat2, family = binomial(link = "logit"), control = list(maxit
= 10000))
gls_y <- c(as.matrix(dat.temp2[, 5:7]))
X1 <- model.matrix(lm(iso_1 ~ a1 + sg_1 + age + race_cat_new + insur_new + edu_cat_new +
bmi_cat_new, dat = dat2))
X2 <- model.matrix(lm(iso_2 ~ aavg_2 + sg_2 + age + race_cat_new + insur_new + edu_cat_new
+ bmi_cat_new, dat = dat2))
X3 <- model.matrix(lm(iso_3 ~ aavg + sg_3 + age + race_cat_new + insur_new + edu_cat_new
+ bmi_cat_new, dat = dat2))
gls_x <- as.matrix(bdiag(X1, X2, X3)) gls_weight <- rep(dat.temp2$weight, 3)
gls_id <- as.factor(rep(1:dim(dat.temp2)[1], 3))
mod_gls <- gls(gls_y ~ -1 + gls_x, weights = varFixed(~ gls_weight), correlation = corSymm(form
= ~ 1 | gls_id))
sigma2 <- rep(1, 3)%*%(cov2cor(getVarCov(mod_gls))*summary(mod_gls)$sigma^2)%*%rep(1, 3)
for(k in 1:nboot) {
dat.boot <- dat.temp2[sample(1:dim(dat.temp2)[1], replace = TRUE), ] dat.boot$weight <-
dat.boot$weight / sum(dat.boot$weight) * dim(dat.boot) [1]
mod.y <- glm(PRETERM ~ asum + isosum + sg_avg + age + race_cat_new+ insur_new + edu_cat_new
+ bmi_cat_new, dat = dat.boot, family = binomial(link = "logit"), control = list(maxit =
10000))
mod.y.int <- glm(PRETERM ~ asum + isosum + asum*isosum + sg_avg + age + race_cat_new + insur_new
+ edu_cat_new + bmi_cat_new, dat = dat.boot, family = binomial(link = "logit"), control
= list(maxit = 10000))
mod.m3 <- lm(iso_3 ~ aavg + sg_3 + age + race_cat_new + insur_new + edu_cat_new + bmi_cat_new,
dat = dat.boot, weights = weight)
mod.m2 <- lm(iso_2 ~ aavg_2 + sg_2 + age + race_cat_new + insur_new + edu_cat_new + bmi_cat_new,
dat = dat.boot, weights = weight)
mod.m1 <- lm(iso_1 ~ a1 + sg_1 + age + race_cat_new + insur_new + edu_cat_new + bmi_cat_new,
dat = dat.boot, weights = weight)
gls_y <- c(as.matrix(dat.boot[, 5:7]))
X1 <- model.matrix(lm(iso_1 ~ a1 + sg_1 + age + race_cat_new + insur_new + edu_cat_new +
bmi_cat_new, dat = dat.boot))
X2 <- model.matrix(lm(iso_2 ~ aavg_2 + sg_2 + age + race_cat_new + insur_new + edu_cat_new
+ bmi_cat_new, dat = dat.boot))
X3 <- model.matrix(lm(iso_3 ~ aavg + sg_3 + age + race_cat_new + insur_new + edu_cat_new
+ bmi_cat_new, dat = dat.boot))
```

```
gls_x <- as.matrix(bdiag(X1, X2, X3))
gls_weight <- rep(dat.boot$weight, 3)
gls_id <- as.factor(rep(1:dim(dat.boot)[1], 3))
mod_gls <- gls(gls_y ~ -1 + gls_x, weights = varFixed(~ gls_weight), correlation = corSymm(form
= ~ 1 | gls_id))
sigma2.boot[i, k] <- rep(1, 3)%*% (cov2cor(getVarCov(mod_gls))*summary(mod_gls)$sigma^2)%*%rep(1,
3)
sg_mean_boot[i, 1, k] <- mean(dat.boot$sg_1)
sg_mean_boot[i, 2, k] <- mean(dat.boot$sg_2)
sg_mean_boot[i, 3, k] <- mean(dat.boot$sg_3)
age_mean_boot[i, k] <- mean(dat.boot$age)
coef.boot[i, 1, k] <- coef(mod.y)[2]
coef.boot[i, 2, k] <- coef(mod.y)[3]
coef.boot[i, 3, k] <- coef(mod.m1)[2]
coef.boot[i, 4, k] <- coef(mod.m2)[2]
coef.boot[i, 5, k] <- coef(mod.m3)[2]
coef.boot[i, 6, k] <- coef(mod.y.int)[2]
coef.boot[i, 7, k] <- coef(mod.y.int)[3]
coef.boot[i, 8, k] <- coef(mod.y.int)[14]
coef.boot[i, 9, k] <- coef(mod.m1)[1]
coef.boot[i, 10, k] <- coef(mod.m2)[1]
coef.boot[i, 11, k] <- coef(mod.m3)[1]
coef.boot[i, 12, k] <- coef(mod.m1)[3]
coef.boot[i, 13, k] <- coef(mod.m2)[3]
coef.boot[i, 14, k] <- coef(mod.m3)[3]
coef.boot[i, 15, k] <- coef(mod.m1)[4]
coef.boot[i, 16, k] <- coef(mod.m2)[4]
coef.boot[i, 17, k] <- coef(mod.m3)[4]
coef.boot[i, 18, k] <- summary(mod_gls)$tTable[2, 1]
coef.boot[i, 19, k] <- summary(mod_gls)$tTable[14, 1]
coef.boot[i, 20, k] <- summary(mod_gls)$tTable[26, 1]
coef.boot[i, 21, k] <- summary(mod_gls)$tTable[1, 1]
coef.boot[i, 22, k] <- summary(mod_gls)$tTable[13, 1]
coef.boot[i, 23, k] <- summary(mod_gls)$tTable[25, 1]
coef.boot[i, 24, k] <- summary(mod_gls)$tTable[3, 1]
coef.boot[i, 25, k] <- summary(mod_gls)$tTable[15, 1]
coef.boot[i, 26, k] <- summary(mod_gls)$tTable[27, 1]
coef.boot[i, 27, k] <- summary(mod_gls)$tTable[4, 1]
coef.boot[i, 28, k] <- summary(mod_gls)$tTable[16, 1]
coef.boot[i, 29, k] <- summary(mod_gls)$tTable[28, 1]
}
eff.tab[, 1] <- 3*coef.tab[, 1] # direct effect for method 3
eff.tab[, 2] <- coef.tab[, 2]*(coef.tab[, 3] + coef.tab[, 4] + coef.tab[, 5]) # indirect
effect for method 3
eff.tab[, 3] <- eff.tab[, 2] / (eff.tab[, 1] + eff.tab[, 2]) # total effect for method 3
eff.tab[, 4] <- 3*(coef.tab[, 6] + coef.tab[, 8]*(sigma2*coef.tab[, 7] - apply(coef.tab[,
18:20], 1, sum)+ apply(coef.tab[, 21:23], 1, sum)+ mean(dat1$sg_1)*coef.tab[, 24] + mean(dat1$sg_2)*
25]+ mean(dat1$sg_3)*coef.tab[, 26] + mean(dat1$age)*apply(coef.tab[, 27:29], 1, sum)))
-9/2*sigma2*coef.tab[, 8]^2 # direct effect for method 4
eff.tab[, 5] <- coef.tab[, 7]*apply(coef.tab[, 18:20], 1, sum) # indirect effect for method
4
eff.tab[, 6] <- eff.tab[, 5] / (eff.tab[, 4] + eff.tab[, 5]) # total effect for method 4
eff.tab[, 7] <- (eff.tab[, 1] + eff.tab[, 2]) # proportion mediated for method 3
eff.tab[, 8] <- (eff.tab[, 4] + eff.tab[, 5]) # proportion mediated for method 4
eff.boot[, 1, ] <- 3*coef.boot[, 1, ]
eff.boot[, 2, ] <- coef.boot[, 2, ]*(coef.boot[, 3, ] + coef.boot[, 4, ] + coef.boot[, 5,
])
eff.boot[, 3, ] <- eff.boot[, 2, ] / (eff.boot[, 1, ] + eff.boot[, 2, ])
```

```
eff.boot[, 4, ] <- 3*(coef.boot[, 6, ] + coef.boot[, 8, ]*(sigma2.boot*coef.boot[, 7, ]
- apply(coef.boot[, 18:20, ], c(1, 3), sum)+ apply(coef.boot[, 21:23, ], c(1, 3), sum) +
sg_mean_boot[, 1, ]*coef.boot[, 24, ] + sg_mean_boot[, 2, ]*coef.boot[, 25, ] + sg_mean_boot[,
3, ]*coef.boot[, 26, ] + age_mean_boot*apply(coef.boot[, 27:29, ], c(1, 3), sum)))
-9/2*sigma2.boot*coef.boot[, 8, ]^2
eff.boot[, 5, ] <- coef.boot[, 7, ]*apply(coef.boot[, 18:20, ], c(1, 3), sum)
eff.boot[, 6, ] <- eff.boot[, 5, ] / (eff.boot[, 4, ] + eff.boot[, 5, ])
eff.boot[, 7, ] <- eff.boot[, 1, ] + eff.boot[, 2, ]
eff.boot[, 8, ] <- eff.boot[, 4, ] + eff.boot[, 5, ]
coef.lower.tab <- apply(coef.boot, 1:2, function(x) x[order(x)][nboot*0.025])
coef.upper.tab <- apply(coef.boot, 1:2, function(x) x[order(x)][nboot*0.975])
eff.lower.tab <- apply(eff.boot, 1:2, function(x) x[order(x)][nboot*0.025])
eff.upper.tab <- apply(eff.boot, 1:2, function(x) x[order(x)][nboot*0.975])
```

References

Vanderweele TJ, Vansteelandt S. 2010. Odds ratios for mediation analysis for a dichotomous outcome. Am J Epidemiol 172:1339-1348.

VanderWeele TJ, Tchetgen Tchetgen E. 2014. Mediation analysis with time-varying exposures and mediators. Available from: http://biostats.bepress.com/harvardbiostat/paper168/ [accessed 01/21/2016].
